# Functional hydrogel empowering 3D printing titanium alloys

**DOI:** 10.1016/j.mtbio.2024.101422

**Published:** 2024-12-24

**Authors:** Weimin Zhang, Jiaxin Zhang, He Liu, Yang Liu, Xiao Sheng, Sixing Zhou, Tiansen Pei, Chen Li, Jincheng Wang

**Affiliations:** a Department of Orthopedics, The Second Hospital of Jilin University, Changchun, 130041, Jilin, China; b Huzhou Central Hospital, Fifth school of Clinical Medical Universtiy, Wuxing, Huzhou, Zhejiang 313000, PR China; c Department of Emergency and Critical Care Medicine, The Second Hospital of Jilin University, Changchun 130041, China

**Keywords:** Porous titanium alloy, Functionalized hydrogel, Local microenvironment, Osseointegration, Drug delivery

## Abstract

Titanium alloys are widely used in the manufacture of orthopedic prosthesis given their excellent mechanical properties and biocompatibility. However, the primary drawbacks of traditional titanium alloy prosthesis are their much higher elastic modulus than cancellous bone and poor interfacial adhesion, which lead to poor osseointegration. 3D-printed porous titanium alloys can partly address these issues, but their bio-inertness still requires modifications to adapt to different physiological and pathological microenvironments. Hydrogels composed of three-dimensional networks of hydrophilic polymers can effectively simulate the extracellular matrix of natural bone and are capable of loading bioactive molecules such as proteins, peptides, growths factors, polysaccharides, or nucleotides for localized release within the human body, by directly participating in biological processes. Combining 3D-printed porous titanium alloys with hydrogels to construct a bioactive composite system that regulates cellular adhesion, proliferation, migration, and differentiation in the local microenvironment is of great significance for enhancing the bioactivity of the prosthesis surface. In this review, we focus on three aspects of the bioactive composite system: (Ⅰ) strategies for constructing bioactive interfaces with hydrogels, and (Ⅱ) how bioactive composite systems regulate the microenvironment under different physiological and pathological conditions to enhance the osteointegration and bone regeneration capability of prostheses. Considering the current research status in this field, innovations in orthopedic prosthesis can be achieved through material optimization, personalized customization, and the development of multifunctional composite systems. These advancements provide essential references for the clinical translation of osseointegration and bone regeneration in various physiological and pathological microenvironments.

## Graphical abstract

**Figure:**
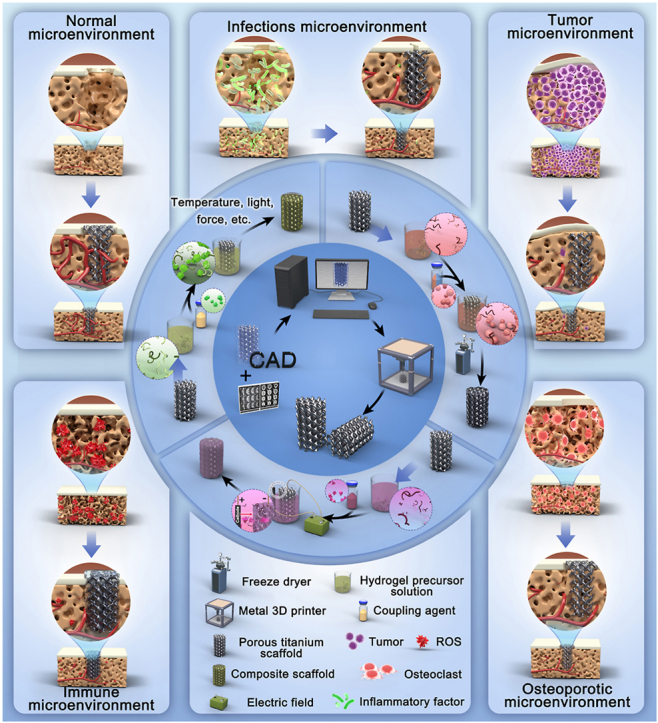

## 1 Introduction

**Fig. 1:**
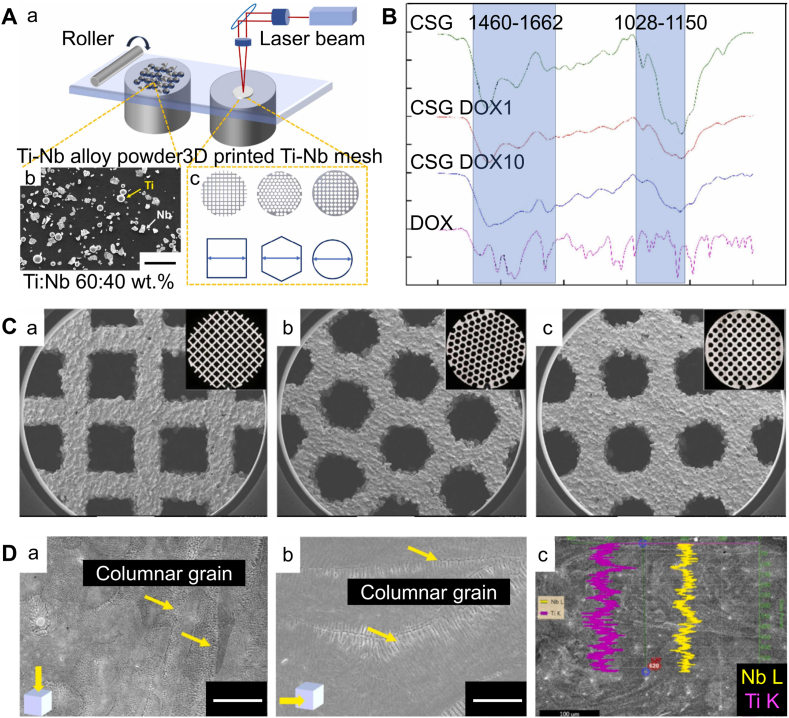
SLM fabrication of 3D-printed porous titanium alloy prosthesis. Reproduced with permission from Ref. [20] (Copyright 2022, Dental Materials). (A) (a) Schematic SLM process. (b) Scanning electron microscope image of titanium-Nb powder (scale bar = 100 μm). (c) Design files and dimensions of the 3D-printed porous titanium alloy scaffold. (B) Fourier transform infrared spectroscopy analysis results of the hydrogel coating. (C) Macroscopic and microscopic morphology of the 3D-printed porous titanium alloy scaffold. (D) (a, b) Top view and side view of the grid microstructure (left image, scale bar = 10 μm; right image, scale bar = 5 μm). (c) EDS line scan and results of the melt pool on the top plane of the grid.

Orthopedic prostheses are widely used to address various clinical needs such as bone defect repair and joint replacement and are continually improving in clinical practice [1]. These prostheses significantly improve patients' quality of life by restoring their mobility and comfort. However, despite technological advancements, about 10 % of prosthesis fail prematurely within the first 10–20 years [2,3]. This failure not only impacts patient health but also increases the burden and resource consumption of the healthcare system. Several thousand patients require revision surgeries annually, which poses a medical challenge and imposes psychological and economic stress on patients and their families. To address this issue, scientists and medical experts are continuously researching and innovating to develop more durable prosthetic materials. Over the past decades, metals have been extensively used in the manufacturing of prosthesis for bone tissue engineering owing to their good mechanical properties [4,5]. Among them, titanium alloys have become one of the optimal choices for prosthesis manufacturing in clinical settings given their excellent biocompatibility and mechanical properties [[6], [7], [8]]. However, the primary disadvantage of the traditional titanium alloy prosthesis is inadequate osseointegration caused by an elastic modulus far higher than that of cancellous bone [9,10]. Osseointegration refers to the direct contact and firm mechanical bonding between the prosthesis and the surrounding bone tissue [11]. 3D printing, also known as additive manufacturing (AM), is a technology based on layer-by-layer manufacturing principles that accumulates materials in a layer-by-layer fashion (Fig. 1A) [12]. This technology can rapidly produce parts of any complex shape through computer-controlled solid modeling based on computer-aided design models or computed tomography scans [13]. AM technology is highly automated, accurate, and repeatable, and therefore widely used in producing tissue-engineered prostheses [14]. With the advancement of 3D printing technologies, such as Powder Bed Fusion and Directed Energy Deposition, the issues associated with traditional titanium alloy prostheses have been effectively resolved. 3D-printed prosthesis offer personalized external geometries that can precisely match bone defects and enable accurate anatomical reconstruction [15]. Additionally, 3D-printed titanium alloy prostheses possess unique controllable porous structures (Fig. 1C and D), giving them an elastic modulus similar to cancellous bone, thus avoiding stress shielding and bone resorption [16]. The ordered porous structure increases the contact area between the prosthesis and host bone, aiding in bone ingrowth and long-term stability, and providing space for loading cells, drugs, and growth factors (Fig. 1B) [[17], [18], [19]].

However, the bio-inertness of titanium alloy materials (i.e., their lack of significant chemical or immune interactions with surrounding tissues) results in a lack of sufficient functional groups on their surface to promote protein and cell adhesion, which cannot induce sufficient bone regeneration, limiting their promotion of bone ingrowth and osseointegration [21]. Moreover, in various pathological microenvironments such as infections, osteoporosis, bone tumors, and immune-related diseases, various factors may impair osteoblast (OB) function around the bone-prosthesis interface, leading to poor bone-prosthesis osseointegration [[22], [23], [24]]. To address these issues, contemporary researchers often adopt surface modification strategies to improve the biological performance of titanium alloy prostheses. Surface modification techniques such as chemical surface modification, physical surface modification, and biological surface modification, play a crucial role in enhancing the physicochemical properties of titanium alloy materials, thereby accelerating bone formation and strengthening chemical bonding [[25], [26], [27]]. These modification techniques not only effectively improve the surface characteristics of titanium materials but also significantly enhance their biocompatibility.

Biological surface-modification techniques have become a recent research hotspot. Biological surface modification involves combining organic bioactive substances such as proteins with the prosthesis surface through electrostatic interactions and hydrogen bonds [28]. Compared to chemical and physical surface modifications, biological surface modification techniques significantly enhance the bioactivity of materials. These methods enable the directed regulation of cellular behavior, promoting cell attachment, proliferation, and bone formation. Additionally, biological modification facilitates the integration of materials with surrounding tissues, reduces the risk of immune rejection, and accelerates bone healing and regeneration by releasing specific biological signals [29]. Among these techniques, hydrogels are one of the most commonly used biological surface-modification techniques and have been widely applied in the surface modification of titanium alloy prostheses [30].Hydrogels are three-dimensional networks composed of hydrophilic polymers, which can well mimic the extracellular matrix (ECM) of natural bone, providing an environment conducive to the adhesion, proliferation, and differentiation of OB and bone cells, thus addressing the bio-inertness of titanium alloy prostheses [31].

Hydrogels can be classified into natural hydrogels and synthetic hydrogels based on the source of their polymer substrates [32]. Natural hydrogels possess good biocompatibility and biodegradability owing to their structure being similar to the ECM of natural tissues and are thus widely used in the biomedical field [33,34]. Synthetic hydrogels, which are renewable materials with highly controllable chemical and physical properties, are widely used in drug delivery, tissue engineering, and biosensing and mainly include alcohols, acrylic acids, and their derivatives [35]. Additionally, synthetic hydrogels possess good chemical reproducibility and their properties are adjustable according to the actual needs [36].

Utilizing hydrogels in conjunction with 3D-printed porous titanium alloy prostheses to construct biological functional composite systems can further improve bone repair and prosthesis interface osseointegration capabilities. Moreover, 3D-printed porous titanium alloy prosthesis modified with hydrogels loaded with different functional components can adapt to various pathological and physiological microenvironments, targeting and addressing various factors leading to insufficient osteogenic capacity, which is crucial for promoting the long-term stability of joint prosthesis interfaces post-replacement surgery [29,37]. Therefore, since 2019, our team has conducted extensive research in this field (Table 1). We have explored the effectiveness of the porous titanium alloy and hydrogel composite systems with bioactive capabilities in promoting bone-prosthesis interface osseointegration in normal physiological environments, osteoporotic environments, and immunopathological environments. These studies aim to evaluate the potential applications of the composite systems under different pathological conditions. The results indicate that the bioactive composite system, composed of rigid 3D-printed porous titanium alloy and soft hydrogel, can mimic the microstructure, mechanical properties, and extracellular matrix microenvironment of natural cancellous bone, thereby promoting the proliferation, adhesion, and differentiation of osteogenic-related cells. Additionally, functionalized hydrogels designed for different prosthetic-surrounding microenvironments can improve the local microenvironment, facilitating bone regeneration and osseointegration at the bone-prosthesis interface.

**Table 1:** Our team's exploration of 3D-printed porous titanium alloy and hydrogel composite systems in various local microenvironments. Reproduced with permission from Refs. [[38], [39], [40], [41], [42], [43], [44], [45], [46], [47]] (All the above content has been authorized by the copyright company and the references are arranged in the order of the images).

Local microenvironment	Matrix/Carrier	Functional components	Application	Scheme	Ref.
Normal microenvironment	Functionalized biomimetic sodium alginate mineralized collagen hydrogels	VEGF	Promote osteogenesis		[38]
			Promote angiogenesis		
	CEC, HA-ALD stiffness-variable hydrogels	–	Promote osteogenesis		[39]
Osteoporotic microenvironment	Thermosensitive poloxamer 407 hydrogels	ZOL	Inhibit osteoclast		[40]
			Promote osteogenesis		
	CEC, ADH, HALD self-healing supramolecular hydrogels	BMSCs	Promote osteogenesis		[41]
		BMP-2			
	Thermosensitive poloxamer 407 hydrogels	OPG	Inhibit osteoclast		[42]
		BMP-2	Promote osteogenesis		
	Thermosensitive poloxamer 407 hydrogels	99 Tc-MDP	Inhibit osteoclast		[43]
			Promote osteogenesis		
	PVA, CEC, agarose and silver nanowires self-healing supramolecular hydrogels	Rapamycin	Improve autophagy in endogenous OP-BMSCs		[44]
Immune microenvironment	CEC, HA-ALD, ADH self-healing supramolecular hydrogels	BMSCs	Inhibit inflammatory cytokines		[45]
			Rebuilds damaged cartilage		
	HA-HYD, HALD, infliximab drug-based hydrogels	Infliximab	Inhibit inflammatory cytokines		[46]
		BMSCs	Rebuilds damaged cartilage		
	ε-PLE@MnCoO, HA-HYD, HALD nanozyme-reinforced hydrogel	ε-PLE@MnCoO	Inhibit inflammatory cytokines		[47]
		BMSCs	Promote osteogenesis		

^a^ N-carboxyethyl chitosan, CEC, b Oxidized hyaluronic acid, HA-ALD, ^c^ Adipic acid dihydrazide, ADH, ^d^ Hyaluronic acid-aldehyde, HALD, ^e^ Polyvinyl alcohol, PVA, ^f^ Hyaluronic acid-hydrazide, HA-HYD.

**Scheme 1:**
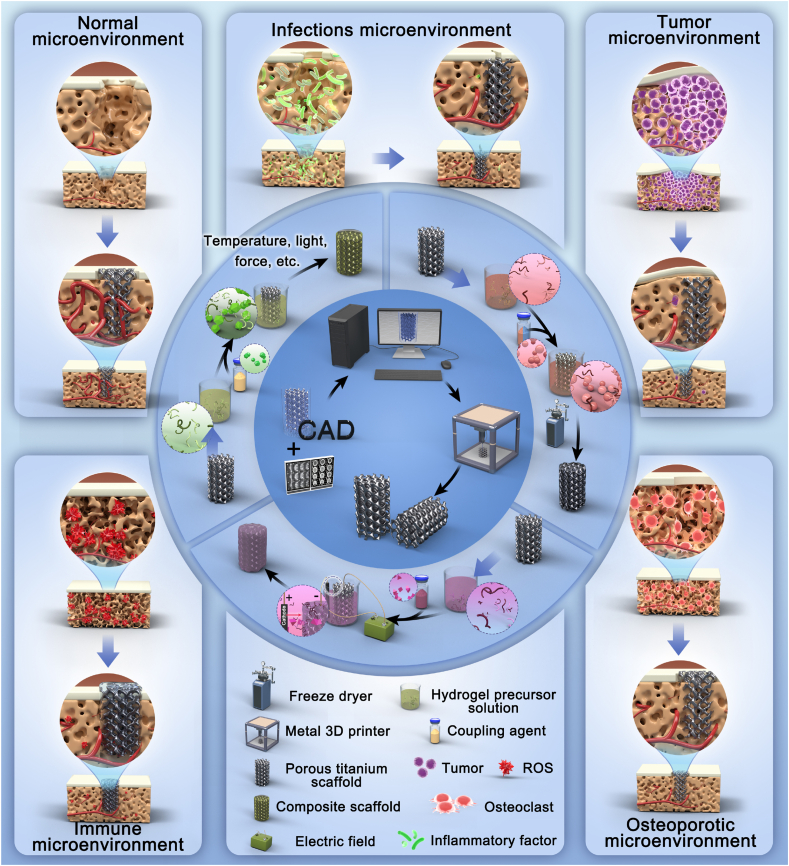
Functionalized hydrogel system enhances osteointegration at the interface of 3D-printed porous titanium alloy prosthesis in various local microenvironments.

This paper reviews the current research progress in promoting prosthesis interface osseointegration based on 3D-printed porous titanium alloy prostheses combined with hydrogel composite systems, to introduce the strategies for constructing bioactive interfaces with hydrogels with a focus on the promotion of bone-prosthesis interface osseointegration by 3D-printed porous titanium alloy hydrogel composite systems under different physiological and pathological conditions, thereby elucidating the effects of functionalized hydrogel systems in enhancing the bioactivity of titanium alloy prosthesis and providing references for clinical translation in the field of prosthesis interface osseointegration and bone regeneration (Scheme 1).

## 2 Strategies for constructing bioactive interfaces with hydrogels

Constructing a 3D-printed porous titanium alloy hydrogel system requires effectively combining the "hard" porous titanium alloy prosthesis with "soft" hydrogels to sufficiently fill hydrogels within the porous titanium alloy prosthesis. Common methods for combining 3D-printed porous titanium alloy prosthesis with hydrogels include the sol-gel method, electrochemical methods, and freeze-drying methods.

### 2.1 Sol-gel method

**Fig. 2:**
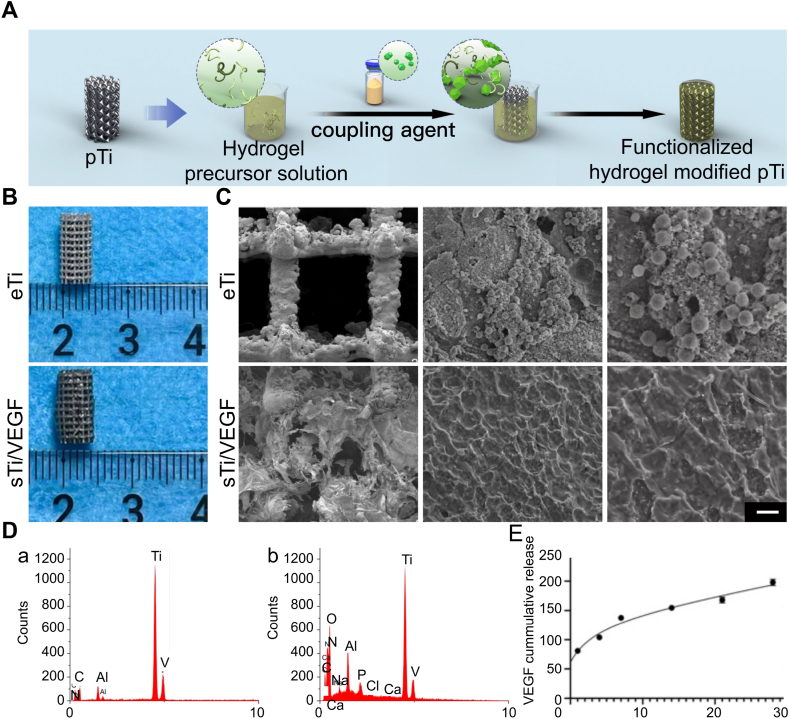
Sol-gel method. Reproduced with permission from Ref. [38] (Copyright 2024, Materials Today Bio) (A) Schematic of the sol-gel method for preparing hydrogel coatings on the surface of 3D-printed porous titanium alloy scaffolds. (B) External appearance of empty titanium (eTi) and FMC-loaded titanium (sTi/VEGF). (C) Microstructure of empty titanium (eTi) and FMC-loaded titanium (sTi/VEGF) (left image, scale bar = 300 μm; middle image, scale bar = 15 μm; right image, scale bar = 8 μm). (D) Energy-dispersive X-ray spectroscopy (EDS) of eTi and sTi/VEGF (a, b). (E) the degradation rate curve of FMC *in vivo* over 28 days (c).

The sol-gel method is a common method for combining 3D-printed porous titanium alloy prosthesis with hydrogels [38]. Placing the 3D-printed porous titanium alloy prosthesis in a precursor solution of the hydrogel, followed by adding a coupling agent to the mixed system to cross-link the non-crosslinked polymer chains into a polymer network, connects the complementary functional groups on the surface of the 3D-printed porous titanium alloy prosthesis (Fig. 2A). Additionally, the coupling agent cross-links with the non-crosslinked polymer chains to form a polymer network, requiring a certain time for cross-linking [48]. Therefore, during the transition from the sol state to the gel state, the mixed system in the sol state can be injected into the 3D-printed porous titanium alloy prosthesis, thereby achieving the combination of the hydrogel in the gel state with the 3D-printed porous titanium alloy prosthesis (Fig. 2B–E). Such hydrogels are mostly stimulus-responsive hydrogels that are a type of porous structural filling material that undergo reversible or irreversible phase transitions *in situ* in response to external stimuli such as light, heat, magnetic fields, or mechanical forces. The stimuli-responsive hydrogels discussed in this review mainly include temperature-responsive and light-responsive hydrogels.

**Fig. 3:**
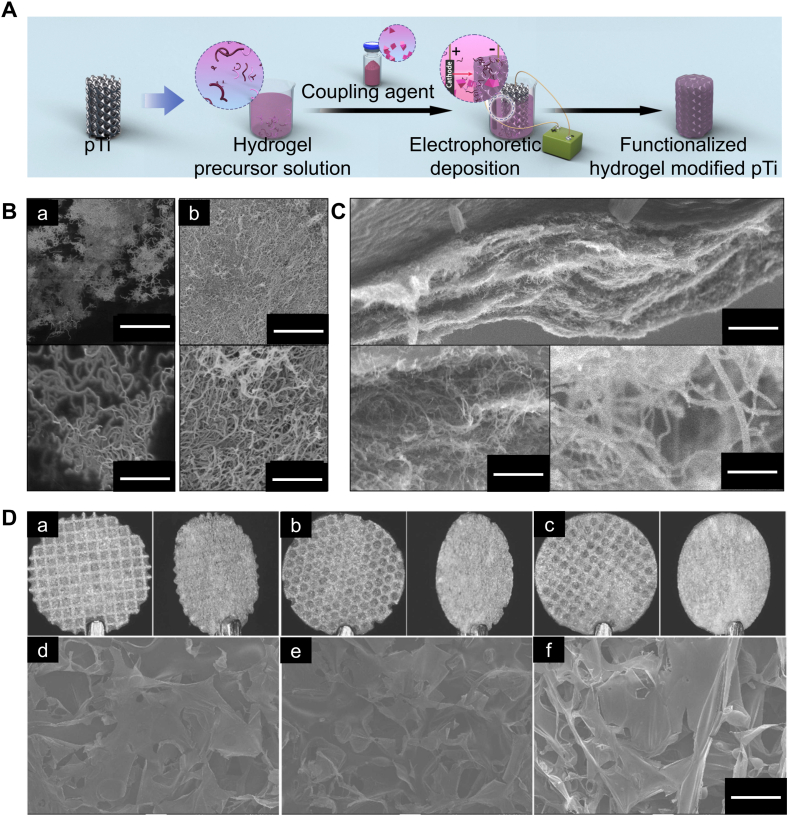
Electrochemical method. (A) Schematic of the electrochemical method for preparing hydrogel coatings on the surface of 3D-printed porous titanium alloy scaffolds. (B) Microstructure of the hydrogel coating (upper image, scale bar = 1 μm; lower image, scale bar = 200 nm). (C) Microstructure of the cross-section of the hydrogel coating (upper image, scale bar = 1 μm; lower left image, scale bar = 500 nm; lower right image, scale bar = 100 nm). Reproduced with permission from Ref. [60] (Copyright 2017, Composites Science and Technology) (D) Macroscopic appearance and microstructure of the hydrogel coating on the surface of the 3D-printed porous titanium alloy scaffold (scale bar = 100 μm). Reproduced with permission from Ref. [20] (Copyright 2022, Dental Materials).

#### 2.1.1 Light-responsive hydrogels

Light-responsive hydrogels are made by introducing light-sensitive groups into thermosensitive or pH-sensitive materials and can be divided into visible light-sensitive and ultraviolet light-sensitive types. Based on their response mechanisms, light-sensitive hydrogels can be classified into three types: those with special photosensitive molecules; those that use ions generated by photosensitive compounds decomposing under light to change the ion concentration difference inside and outside the gel, causing osmotic pressure mutations in the gel and thus achieving responsiveness; and those that introduce chromophores that change the physicochemical properties of the polymer under light exposure [49]. Moreover, the phase change of light-responsive hydrogels can achieve rapid gelation and *in situ* injection. Therefore, the precursor solution of light-responsive hydrogels mixed with a photoinitiator can be pre-injected into alkali-heat-treated 3D-printed porous titanium alloy prosthesis [50]. Then, through specific ultraviolet light exposure, rapid gelation of the hydrogel can be achieved, by combining it with an alkali-heat-treated 3D-printed porous titanium alloy prosthesis. Through precise spatiotemporal control, light-responsive hydrogels can modulate the release of encapsulated drugs/proteins and activate cell behavior, making them widely applicable in biomedical fields [[51], [52], [53]]. However, the bonding strength between the "soft" hydrogel and "hard" porous titanium alloy prosthesis is another issue, as low bonding strength between them can lead to early separation of the hydrogel from the 3D-printed porous titanium alloy prosthesis post-prosthesisation. Zhao et al. [54] proposed an *in situ* mineralization method by introducing CaCO_3_ particles into UV-responsive chitosan (CS) hydrogels and filling them into the internal pores of titanium prosthesis to modify the entire porous system, improving the bonding strength on the hydrogel-porous titanium alloy prosthesis interface. The UV-responsive CS hydrogels mineralized with CaCO_3_ particles can provide a biomimetic environment for bone marrow mesenchymal stem cells (BMSCS) osteogenesis and form direct integration between the host bone and bioactive minerals, thus accelerating bone healing [55].

#### 2.1.2 Temperature-responsive hydrogels

Temperature-responsive hydrogels, also known as thermosensitive hydrogels, are a type of hydrogels sensitive to temperature changes. When the temperature exceeds the lower critical solution temperature, the network structure inside the hydrogel changes accordingly, transitioning the hydrogel from a sol state to a gel state [56]. Therefore, placing 3D-printed porous titanium alloy prosthesis in thermosensitive hydrogels and changing the temperature can achieve an effective combination of the hydrogel and porous titanium alloy prosthesis. This temperature sensitivity allows thermosensitive hydrogels to be widely used in drug delivery [57]. Thermosensitive hydrogels encapsulating various bioactive substances transition from a sol state to a gel state in response to temperature changes, enabling them to carry drugs *in vitro* and release them *in vivo*. The slow and sustained degradation process allows the encapsulated substances to be released slowly in the body, maintaining their effective concentration for an extended period [58].

### 2.2 Electrochemical methods

Electrochemical methods mainly include electrochemical deposition and electrophoretic deposition. Electrochemical deposition uses a constant current deposition mode to control current density, with surface-treated 3D-printed porous titanium alloy prosthesis acting as one electrode for electrochemical reactions, and the electrolyte being a hydrogel precursor solution, producing hydrogel coatings combined with 3D-printed porous titanium alloy prosthesis under specific temperature and pH conditions. Hydrogel coatings prepared by electrochemical deposition are suitable for porous titanium alloy prosthesis of different morphologies and can uniformly coat the surface of 3D-printed porous titanium alloy prosthesis.

Electrophoretic deposition is the process of depositing colloidal particles into a material in a stable suspension through the application of a direct-current electric field. Specifically, when a direct voltage is applied, charged particles dispersed in the medium solvent move to the cathode, forming an insoluble substance on the cathode surface and get deposited on the working surface (Fig. 3A) [59]. It is a simple and direct method for preparing hydrogel (Fig. 3B and C) [60]. Electrophoretic deposition can uniformly deposit organic, inorganic, and composite coatings on 3D-printed porous titanium alloy prosthesis [61]. Research has been conducted on using electrophoretic deposition to manufacture hydrogel coatings on the surface of 3D-printed porous titanium alloy prosthesis (Fig. 3D) [20]. Moreover, the thickness of the hydrogel deposited on the surface of titanium alloy prosthesis can be controlled by the electric field strength and deposition time [60].

### 2.3 Freeze-drying method

**Fig. 4:**
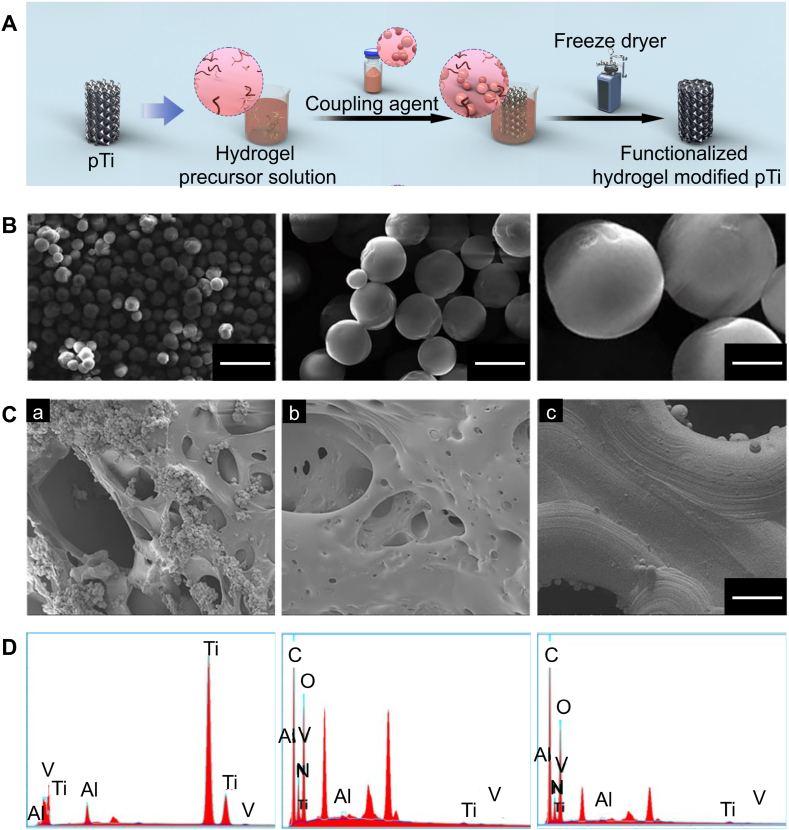
Freeze-drying method. Reproduced with permission from Ref. [65] (Copyright 2022, Frontiers in Bioengineering and Biotechnology). (A) Schematic of the freeze-drying method for preparing hydrogel coatings on the surface of 3D-printed porous titanium alloy scaffolds. (B) Scanning electron microscope images of core-shell microspheres. (C) 3D-printed titanium alloy porous scaffold with dual-factor loaded core-shell microsphere-gelatin coating (a), 3D-printed titanium alloy porous scaffold with gelatin coating (b), and microstructure of the 3D-printed titanium alloy porous scaffold (c). (D) 3D-printed titanium alloy porous scaffold with dual-factor loaded core-shell microsphere-gelatin coating, 3D-printed titanium alloy porous scaffold with gelatin coating, and EDS analysis results of the 3D-printed titanium alloy porous scaffold.

The freeze-drying method is a technique for preparing 3D porous materials by freezing the precursor solution composed of liquid solvent and solute particles into a solid state at low temperatures. This method involves two stages: the freezing stage of the precursor solution at 0 °C and the thawing stage at room temperature, primarily aimed at controlling the ice crystallization process (freezing) and the formation of ordered structures (thawing) to achieve optimal performance of the hydrogel [62]. Under low temperature and low-pressure conditions, the solvent crystals sublime into gas to remove the organic solvent, resulting in a highly porous material [63]. Moreover, during the freeze-drying process of preparing hydrogels, the initial components in the precursor solution can be uniformly dispersed in the initial suspension or solution, usually without differential separation during freezing, resulting in highly uniform hydrogel porous materials. Thus, the freeze-drying method can be used to combine hydrogels with 3D-printed porous titanium alloy prosthesis [64]. For example, Liu et al. [65] used the freeze-drying method to place 3D-printed hollow hexagonal titanium alloy porous prosthesis in precursor solutions composed of VEGF/BMP-2-loaded core-shell microspheres and gelatin, pre-freezing at −80 °C, followed by freeze-drying for 72 h, and stirring with 1 % genipin as a cross-linking agent at −37 °C for 12 h, thereby combining 3D-printed porous titanium alloy prosthesis with hydrogels to construct a 3D-printed porous titanium alloy prosthesis-loaded VEGF/BMP-2 core-shell microsphere sustained-release system (Fig. 4A). This approach enables the uniform distribution of core-shell microspheres within the hydrogel (Fig. 4B and C), and the hydrogel can be evenly distributed on the surface of the 3D-printed titanium alloy scaffold (Fig. 4D).

## 3 Functionalized hydrogel prosthesis to promote bone regeneration and osseointegration

Prosthesis loosening, peri-prosthetic fractures, and other complications are common after arthroplasty and are usually related to insufficient osteogenic capacity caused by various factors. The local microenvironment around the prosthesis can be divided into the following five categories: (Ⅰ) Normal physiological microenvironment; (Ⅱ) Infectious pathological microenvironment; (Ⅲ) Osteoporotic pathological microenvironment; (Ⅳ) Tumorous pathological microenvironment; and (Ⅴ) Immune pathological microenvironment. In recent years, the application of titanium alloys combined with hydrogels in promoting prosthesis interface osseointegration has become increasingly common given their many advantages. In response to different pathological microenvironments, researchers have loaded cells, drugs, growth factors, metal ions, and other substances into hydrogels and 3D-printed porous titanium alloy prosthesis to construct functionalized composite systems, with the aim to promote bone generation around the prosthesis. In subsequent sections, we will discuss the relevant progress of functionalized hydrogel systems in promoting osseointegration of 3D-printed porous titanium alloy prosthesis from the perspective of the prosthesis-surrounding microenvironment.

### 3.1 Normal physiological microenvironment

The normal physiological microenvironment refers to the stable physiological state of cells, tissues, and organs within the human body, including appropriate temperature, pH, oxygen, and nutrient concentrations, along with maintenance of electrolyte balance, immune function, and water stability [66,67]. In this environment, various biochemical processes can proceed normally, helping to maintain healthy cell and tissue functions [68]. However, because of the bio-inertness of titanium alloys, some patients may have insufficient bone regeneration capacity under normal physiological conditions, preventing firm osseointegration at the bone-prosthesis interface. Therefore, modifying the prosthesis to prevent complications such as prosthesis loosening, subsidence, and peri-prosthetic fractures after arthroplasty is essential. In the following sections, we describe in detail the applications of functionalized hydrogel modified porous titanium alloy prosthesis loaded with different components to promote bone regeneration and osseointegration.

#### 3.1.1 Angiogenesis-promoting hydrogels

**Fig. 5:**
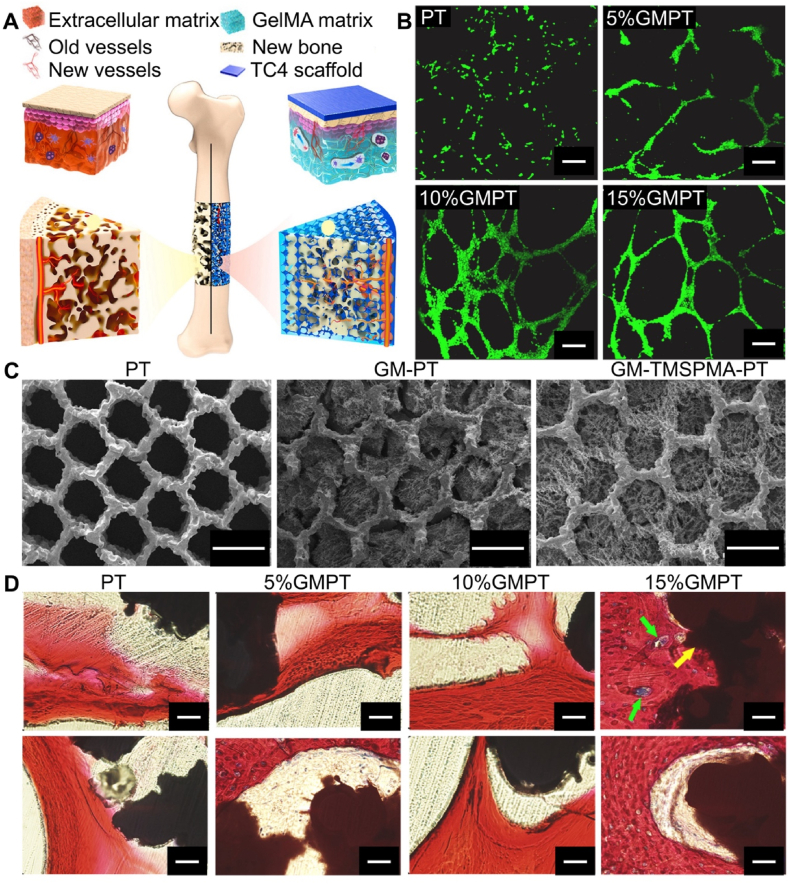
Normal physiological microenvironment. Reproduced with permission from Ref. [70] (Copyright 2021, Bioactive Materials). (A) Schematic of the biomimetic GMPT with dual biomimetic functions for treating bone defects in a physiological microenvironment. (B) Immunofluorescence of vascular network formation (scale bar = 200 μm). (C) Microstructure of GelMA and PT scaffolds in PT, GM-PT, and GM-TMSPMA-PT (scale bar = 500 μm). (D) Van Gieson staining results of the prosthesis specimens in the rabbit radial defect area at 12 weeks (upper row of images, scale bar = 200 μm; lower row of images, scale bar = 50 μm).

Gelatin methacryloyl (GelMA) is a hydrogel material generated by reacting gelatin with methacryloyl chloride. It is widely used in biomedicine and tissue engineering and has excellent biocompatibility, controllable mechanical properties, and biodegradability, and its chemical structure and mechanical properties can be adjusted by regulating its concentration and light cross-linking strength, which is suitable for constructing artificial tissues, three-dimensional cell culture, drug delivery, and bioprinting applications. Studies have shown that GelMA can promote angiogenesis, which is crucial for bone regeneration [69]. Despite GelMA's relatively low mechanical strength, making it unsuitable for repairing load-bearing bones, it can be used as a coating for metal prosthesis to improve their mechanical performance deficiencies. Ma et al. [70] combined 3D-printed porous titanium scaffolds (PT), designed to simulate the microstructure and mechanical properties of natural cancellous bone, with soft GelMA hydrogel matrices that mimic the ECM microenvironment (Fig. 5A). This combination—called GMPT—is used for repairing large bone defects. The hydrogel fills the interior of the 3D-printed porous titanium alloy scaffold (Fig. 5C). Compared to pure titanium alloy prosthesis, GelMA hydrogel in the composite system provided a favorable platform for BMSCs adhesion and differentiation, promoting BMSCs proliferation and differentiation inside the scaffold. Studies have shown that when the GelMA concentration was 10 %, cell adhesion, and differentiation capacity were the strongest, with the most significant angiogenesis-promoting ability (Fig. 5B). Histological analysis of the rabbit radial defect area at 4 and 12 weeks post-prosthesisation indicated that in the GMPT group, trabeculae were significantly thicker and more numerous than in the PT group at both 4- and 12-weeks post-surgery (Fig. 5D). *In vivo* experiments also demonstrated that the 10 % GMPT group had the strongest osteogenic and angiogenic capacity.

#### 3.1.2 Inorganic compounds

Calcium phosphate bioceramics are inorganic compounds with compositions similar to bone minerals and closely related to biological reactions occurring during bone remodeling, capable of stimulating bone formation and integration with unique bone substitute properties, and are thus widely used in orthopedics [71,72]. The most extensively studied calcium phosphate bioceramics are hydroxyapatite (HA) and tricalcium phosphate (TCP), which have already been applied clinically [73].

HA is the main inorganic component in bone tissue and is widely used in bone tissue engineering owing to its excellent bioactivity, osteoinductivity, and osteoconductivity [74]. HA can chemically bond with bone tissue, releasing calcium ions (Ca2+) involved in metabolism, stimulating or inducing bone regeneration [75]; furthermore, nano-hydroxyapatite (nHA) can improve bone regeneration by promoting angiogenesis. Additionally, nHA's good biodegradability allows drugs to bind to bone and release slowly [76]. The nanoscale diameter of nHA provides a larger surface area for adsorbing cells, drugs, or other bioactive molecules (e.g., growth factors, antibiotics, and chemotherapy drugs) [73,77]. Due to its targeted delivery and precise drug-release capabilities, nHA is widely used in orthopedics for drug and bioactive component delivery [78,79]. Utilizing nHA coatings on 3D-printed biphasic calcium phosphate (BCP) scaffolds can promote the adhesion, proliferation, and osteogenic and vascular differentiation of peripheral blood-derived mesenchymal stem cells and endothelial progenitor cells on BCP scaffolds [80]. However, HA's poor mechanical properties, slow degradation rate, and single function limit its standalone use as a scaffold material in bone tissue engineering. Thus, researchers began exploring the application of calcium phosphate bioceramics as coatings on metal prosthesis to improve prosthesis performance [76,81]. Specifically, calcium phosphate bioceramics can be applied to metal prosthesis surfaces through coating or sintering methods. This approach can enhance the biocompatibility of metal prosthesis and provide an ideal growth environment for osteoblast proliferation and differentiation. However, these traditional coating methods have issues such as inadequate adhesion and detachment of bioceramics [3]. Some studies have also explored combining hydrogels with calcium phosphate bioceramics. Hydrogels can bond to prosthesis surfaces through electrostatic interactions and hydrogen bonds [28]. Using hydrogels to coat calcium phosphate bioceramics on metal prosthesis can achieve firmer bonding between bioceramics and prosthesis than using traditional coating methods. Composite materials with specific properties can be prepared by combining HA with other materials using hydrogels, to further expand their applications in bone tissue engineering [81]. Kumar et al. [82] designed a hydrogel-modified porous titanium alloy prosthesis loaded with HA and OB for treating critical-sized bone defects, where the uniform distribution of nHA within the porous prosthesis provided an ideal environment for bone mineralization, enhancing the bioactivity of the prosthesis and promoting OB function.

TCP is another common bioceramic bone substitute with good bioactivity and biocompatibility, making it a promising bone scaffold material given its osteogenic capacity through the release of calcium and phosphorus ions. Among them, β-tricalcium phosphate (β-TCP) is a commonly used substitute for autografts in clinical settings. Porous β-TCP has weaker mechanical strength and rapid absorption characteristics than HA, allowing for rapid biodegradation in the body. However, its poor mechanical strength limits its applications in orthopedics. Considering these properties, Kang et al. [83] designed a 3D-printed porous titanium interbody cage loaded with demineralized bone matrix (DBM)/TCP hydrogel to enhance its osteogenic capacity. The TCP coating on the titanium alloy prosthesis through hydrogels addressed TCP's poor mechanical strength, while enhancing the bioactivity of the titanium alloy prosthesis. *In vivo* study results indicated that the Ti/DBM prosthesis containing TCP had better bone regeneration than Ti/DBM prosthesis without TCP. The TCP and DBM loadings provided favorable conditions for osteogenic differentiation, promoting bone formation and effectively inducing osteogenesis in the 3D-printed titanium interbody cage [83]. Therefore, loading calcium phosphate bioceramics into hydrogels to modify 3D-printed porous titanium alloy prosthesis can significantly improve bone-prosthesis interface osseointegration.

##### 3.1.2.1 Growth factors

Bone repair is a highly complex process that requires the coordinated action of multiple components, among which growth factors are a class of polypeptides that can bind to specific, high-affinity cell membrane receptors to regulate cell growth and other functions. Growth factors can act on local OB and OC through autocrine or paracrine mechanisms, stimulating cell division and regulating cell proliferation [84]. Additionally, the bone matrix contains numerous growth factors, including bone morphogenetic proteins (BMPs), transforming growth factor-beta (TGF-β), and platelet-derived growth factors (PDGF) [85]. Imbalances and pathological levels of cytokines are key factors in the development of bone diseases. Therefore, bone regeneration can be achieved by using different bioactive molecules of varying potencies.

###### 3.1.2.1.1 Platelet-derived growth factor family

The platelet-derived growth factor family mainly consists of PDGF and vascular endothelial growth factor (VEGF), with both playing crucial roles in tissue repair. VEGF is an essential growth factor stimulating new blood vessel formation, promoting new blood vessels to form from existing vascular networks, transporting nutrients and oxygen, facilitating cell migration, and maintaining an appropriate metabolic microenvironment [86]. Moreover, VEGF plays a critical role in bone formation and healing by recruiting, maintaining, and promoting the activity of bone-forming cells [87]. Therefore, using VEGF can activate angiogenic factors to coordinate angiogenesis and bone regeneration. Previous reports have indicated that hydrogels loaded with VEGF can induce endothelial cell recruitment and the formation of capillary-like structures, thereby inducing three-dimensional angiogenesis *in vitro* [88]. Li et al. [89] designed a porous titanium alloy prosthesis/thermosensitive collagen hydrogel system loaded with VEGF to promote local bone bonding and angiogenesis. The results showed that the composite prosthesis with VEGF exhibited stronger cell growth and angiogenesis-promoting capabilities than those without VEGF. Mineralized collagen (MC) is the basic unit of natural bone tissue and can induce bone regeneration. However, unmodified MC has poor mechanical properties and a single composition, which is unsuitable for complex physiological environments. Sheng et al. [38] introduced VEGF into MC materials to construct functionalized mineralized collagen (FMC) with good mechanical strength and sustained growth factor release capability. Filling FMC into the pores of 3D-printed titanium alloy scaffolds created a new bio-camouflaging interface. The continuous release of VEGF from the degrading FMC significantly promoted bone and vascular regeneration.

###### 3.1.2.1.2 Bone morphogenetic protein family

BMPs are members of the TGF-β family and promote osteogenesis by stimulating DNA synthesis and cell replication and induce mesenchymal stem cell differentiation into OB [90]. BMPs can facilitate the healing of bone defects and reduce complications, thereby playing a significant role in promoting osteogenesis and chondrogenesis [91]. During bone formation, members of the BMP family primarily act as differentiation factors by inducing a complete cascade reaction of bone formation including mesenchymal stem cell migration and differentiation into OB, which are crucial in bone growth and development and to maintain bone environmental homeostasis [92,93]. However, at the cellular level, the BMP family primarily acts as transforming growth factors promoting mesenchymal stem cell migration and osteogenic differentiation, without promoting cell proliferation [93]. Therefore, using BMP alone to promote bone regeneration and prosthesis interface osseointegration may not achieve optimal results, requiring the combined use with VEGF and FGF to promote cell proliferation and differentiation. Studies have demonstrated that the combined application of VEGF and BMPs not only promotes osteoblast differentiation but also ensures an adequate blood supply and oxygen delivery during bone regeneration through the induction of angiogenesis. Moreover, VEGF enhances the migration of mesenchymal stem cells, while BMPs facilitate their differentiation into osteoblasts, thus further optimizing the efficiency of bone regeneration [94,95]. BMP-9 is a crucial member of the BMP family and a primary mediator of osteogenic differentiation, and is considered the most osteogenic BMP [96]. Che et al. [58] constructed a 3D-printed composite porous titanium prosthesis system loaded with VEGF/BMP-9 thermosensitive collagen hydrogels. Their study results indicated that single VEGF significantly promoted HUVEC migration, chemotaxis, and angiogenesis, while BMP-9 significantly enhanced HUVEC migration and angiogenesis, promoting blood flow reconstruction and osseointegration at bone defects. This might be because of the synergistic effect of VEGF and BMP-9 in promoting osteogenesis and angiogenesis [97]. BMP-9 upregulates HIF-1a expression in MSCs through the SmaD1/5/8 signaling pathway, where HIF-1a is a direct upstream regulator of VEGF that directly induces VEGF synthesis and secretion, thus promoting angiogenesis by inducing angiogenesis and osteogenesis signaling pathways in MSCs [98]. In addition to BMP-9, existing studies have shown that the combination of BMP-2 and VEGF plays a significant role in coordinating osteogenesis and promoting osteogenic differentiation [99]. Liu et al. [65] chose BMP-2/VEGF core-shell microspheres to prepare a gelatin-coated 3D-printed porous titanium alloy, thus creating a titanium alloy microsphere prosthesis release system to repair and reconstruct bone defects. To observe the osteogenic capacity of BMP-2 and VEGF, groups with gelatin-coated 3D-printed porous titanium alloy prosthesis and pure 3D-printed porous titanium alloy prosthesis were set up. The results indicated that composite prosthesis containing BMP-2 and VEGF achieved sequential release of VEGF and BMP-2, promoting OB adhesion, proliferation, and differentiation, enhancing the osseointegration capacity of this composite system *in vivo*.

##### 3.1.2.2 Immune cell factors

Extensive research has shown that the immune system plays a crucial role in tissue healing. Immune cells can directly affect host responses at injury sites and influence related tissue-specific cell populations and the activity of recruited and resident stem cells [100]. Wang et al. [101] prepared a biomimetic polysaccharide hydrogel 3D-printed porous titanium alloy composite prosthesis delivering IL-4 and BMP-2, capable of continuously delivering IL-4 to polarize host macrophages to the M2 phenotype. M2 phenotype macrophages secrete various cytokines, including TGF-β, crucial for promoting tissue healing and regeneration at injury sites [102]. This mixed system exhibits strong angiogenesis capacity, promoting hBMSC proliferation and osteogenic differentiation [103,104]. Additionally, bioinformatics data reveal that IL-4 and BMP-2 can interact through multiple signaling pathways such as the mitogen-activated protein kinase (MAPK) pathway, to achieve synergistic effects on osteogenesis [99]. Moreover, M2-type macrophages promote target cell differentiation and proliferation [103], enhancing cell defense against pathogens, and creating a healing-promoting microenvironment in bone defects [105].

#### 3.1.3 Metal components

In recent years, hybrid hydrogels synthesized based on metal particles with metal ions have demonstrated excellent physicochemical properties, bone sensitivity, and osteogenic potential, offering extensive research prospects in bone tissue engineering [106]. Several studies have confirmed that metal ion-modified composite hydrogel materials effectively promote bone vascularization and regeneration [107,108]. Strontium (Sr) is a crucial trace metal element in the human body that promotes OB proliferation, differentiation, and activity, thereby increasing bone matrix synthesis [109]. Additionally, Sr can enhance the function of soft tissue cells [110], presenting significant potential for treating bone defects and promoting prosthesis interface osseointegration. Strontium promotes osteogenesis through a complex mechanism, simply summarized as promoting OB differentiation and migration through the potential calcium-sensitive receptor pathway, leading to OB proliferation [111]. Additionally, Sr ions have been shown to effectively inhibit the expression of pro-inflammatory cytokines and maintain an anti-inflammatory microenvironment by modulating macrophage phenotypes [112]. Trabecular titanium (TT) is a porous titanium alloy prosthesis manufactured by EBM technology with a structure similar to trabecular bone and is clinically used in acetabular cups and revision components [113]. Lovati et al. [114] constructed a composite system using TT prosthesis preloaded with strontium-enriched amidated carboxymethyl cellulose hydrogel-loaded autologous BMSCs. The *in vitro* study results indicated that the presence of Sr ions significantly increased cell retention within TT and BMSCs osteogenic differentiation, with the composite system exhibiting better matrix deposition capacity in the presence of Sr ions, further enhancing the osseointegration capacity of the composite system. Moreover, the composite system exhibited early angiogenesis capability, playing a vital role in bone regeneration.

Magnesium (Mg) is the fourth-most abundant cation in the human body and plays a crucial role in metabolism [115,116]. Recent studies have suggested that Mg has the potential to promote osseointegration and angiogenesis [117]. The degradation rate in physiological environments once limited the application of Mg-based alloys in bone repair [118]. However, with the development of biomaterials such as alloying and hydrogel surface coating strategies, the degradation of Mg can be delayed. Compared to alloying, hydrogel degradation is controllable, allowing for controlled Mg ion release. Zhang et al. [119] prepared GelMA/TCS/POSS-Mg hydrogels loaded with Mg ions, effectively promoting cell adhesion, spreading, and proliferation. Furthermore, the introduction of Mg2+ stimulated the osteogenic differentiation of bone marrow mesenchymal stem cells and promoted angiogenesis both *in vitro* and *in vivo*, facilitating subsequent bone regeneration in rat cranial defects.

Zinc (Zn) is an essential trace nutrient in the human body that plays a crucial role in many physiological processes, including bone homeostasis. Additionally, Zn is one of the metal ions necessary for bone formation and mineralization, serving as a co-factor for multiple enzymes (e.g., alkaline phosphatase and collagenase), promoting bone metabolism and regeneration in bone matrix [120]. Numerous studies have indicated that Zn possesses good biocompatibility and osteoinductive properties [121]. Zn ions can regulate the expression of osteogenesis-related genes such as runt-related transcription factor 2 (Runx-2), type I collagen, alkaline phosphatase (ALP), and osteocalcin (OCN) [122,123]. Additionally, Zn can promote the migration and differentiation of BMSCs and angiogenesis by activating the MAPK pathway [124]. Lv et al. [125] prepared Zn-containing hydrogels demonstrating significant osteoinductive activity, promoting ALP activity, calcium nodule formation, and osteogenic gene expression. Moreover, Zn ions released from the degrading hydrogels enhanced umbilical vein endothelial cell migration and angiogenesis, exhibiting good osteogenic and angiogenic capabilities *in vivo*.

Adequate angiogenesis is crucial for the reconstruction and regeneration of bone matrix during bone repair [126,127]. New blood vessels provide essential nutrients and cytokines for bone regeneration, regulating angiogenesis and osteogenesis processes [128,129]. Thus, enhancing the angiogenic capacity of bone repair biomaterials is essential for improving bone regeneration success rates. To promote the formation of vascular networks in defect areas, many researchers have added growth factors or cytokines to enhance the angiogenic effect of biomaterials [130]. Compared to these growth factors, metal ions such as copper, cobalt, Mg, Zn, and Sr are inexpensive and readily available, with proven angiogenic activity, and widely used in hydrogels, composite scaffolds, guided tissue regeneration membranes, and other biomaterials for treating bone-related diseases. Additionally, loading multiple metal ions can synergistically promote angiogenesis to some extent and enhance bone tissue formation [131].

#### 3.1.4 Cells

Bone marrow mesenchymal stem cells are a type of multipotent stem cell that play a crucial role in the three stages of osseointegration: secretion of hydrophilic protein membranes to recruit BMSCs, formation of osteoblast progenitors, and secretion and mineralization of the ECM by osteoblasts [[132], [133], [134]]. However, the aging and functional impairment of autologous BMSCs caused by degenerative diseases limit the effectiveness of osseointegration. To address these issues, Wang et al. [39] designed a self-healing supramolecular hydrogel with variable stiffness to encapsulate BMSCs. This hydrogel was combined with a 3D-printed porous titanium alloy to construct a composite system with biomimetic capabilities to enhance osseointegration at the bone-prosthesis interface. The variable stiffness of the hydrogel generates mechanical stimuli that are transduced into intracellular functions through the FAK-MAPK pathway, thereby precisely mediating the osteogenic differentiation of BMSCs. This further accelerates the deposition of bone matrix, promoting early bone ingrowth and osseointegration, both within and around the prosthesis. Bone tissue regeneration is closely linked to vascularization, with bone growth, development, mineralization, remodeling, and tissue regeneration all based on the formation of an excellent vascular network [135]. Chen et al. [69]combined hydrogels loaded with BMSCs and human umbilical vein endothelial cells (HUVECs) with porous titanium alloy prosthesis to create a composite system with strong angiogenesis capabilities. HUVECs possess significant angiogenesis capabilities *in vitro* and are hence widely used in *vitro* vascular systems and angiogenesis research [136]. Their addition enhanced the angiogenesis capacity of porous titanium alloy prosthesis, providing sufficient nutritional support for BMSC proliferation and differentiation, which is crucial for activating OB function and accelerating new bone formation [137]. The composite system significantly improved the coupling of osteogenesis-angiogenesis processes and vascular network regeneration in the early prosthesisation stage, enhancing the osteogenic and angiogenic capacity of porous titanium alloy prosthesis.

EPCs are a pluripotent hematopoietic stem cell subpopulation capable of proliferating, migrating to endothelial injury sites, and differentiating into endothelial cells, thus promoting neovascularization [138]. Previous studies have shown that EPCs can enhance angiogenic activity at fracture sites, accelerating the healing process [139]. Therefore, EPCs are key factors in promoting angiogenesis and accelerating bone formation after bone injury. Zhao et al. [140] chose EPCs as angiogenic components to construct a 3D-printed porous Ti6Al4V prosthesis loaded with EPCs and BMSCs, thus promoting osteogenesis and angiogenesis. Microvascular imaging detection showed that the composite prosthesis significantly induced angiogenesis. At the transcriptional level, the composite prosthesis upregulated the expression of osteogenesis-related genes *ALP* and *OCN* and angiogenesis-related genes *HIF1-α* and *VEGF*, exhibiting excellent osteogenic and angiogenic capacity. Additionally, BMSCs in the mixed system have autocrine and paracrine functions, secreting numerous soluble growth factors, cytokines, and exosomes, benefiting EPC proliferation, migration, and vascular formation [141].

#### 3.1.5 Drugs

**Fig. 6:**
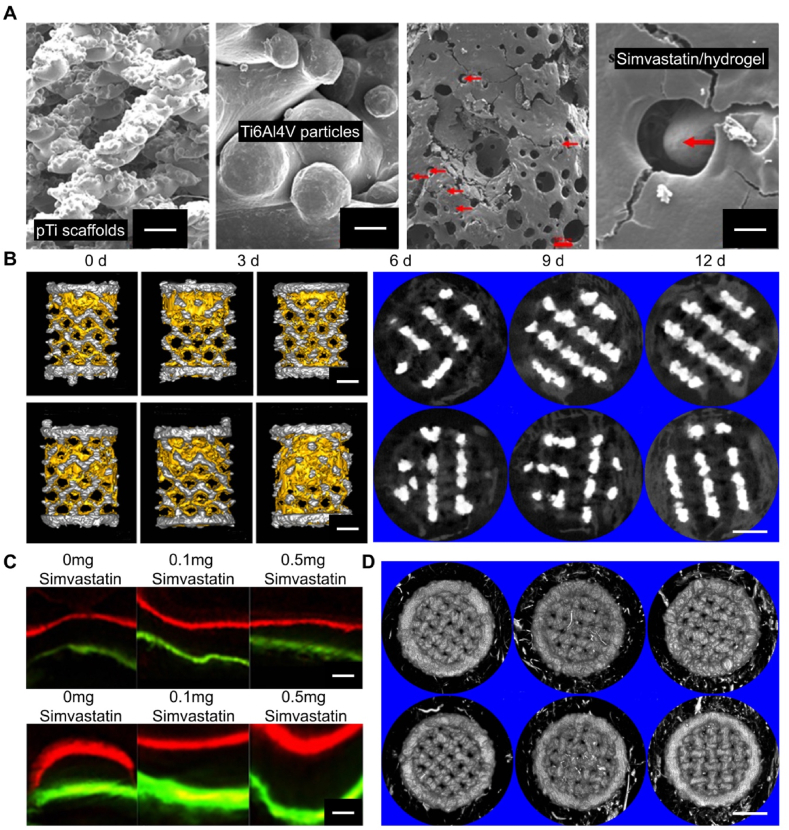
Normal physiological microenvironment. Reproduced with permission from Ref. [144] (Copyright 2016, Biofabrication). (A) Characterization of the 3D-printed porous titanium alloy hydrogel composite system. (B) Representative images of bone ingrowth and osseointegration within/around the pTI scaffold at 4 and 8 weeks post-prosthesisation (scale bar = 1 mm). (C) The dynamic histomorphology of new bone in pTi scaffolds at 4 and 8 weeks post-prosthesisation (scale bar = 20 μm). (D) Representative images of the vascularization within and around the pTI scaffold at 8 weeks post-prosthesisation (scale bar = 1 mm).

Given the importance of angiogenesis for bone tissue repair, many drugs have been discovered to promote angiogenesis. Simvastatin not only enhances ECM differentiation and mineralization in bone cells *in vitro* by stimulating BMP-2 expression in OB [142] but also has angiogenesis-promoting functions both *in vitro* and *in vivo* [135]. However, traditional drug administration methods’ limitations lead to extremely low local bioavailability, failing to meet bone regeneration needs [143]. To address the low local bioavailability of simvastatin, Liu et al. [144] introduced simvastatin into hydrogel systems and combined them with 3D-printed porous titanium alloy prosthesis (Fig. 6A). The mixed system achieved effective local release of simvastatin, significantly promoting angiogenesis and bone ingrowth within and around the prosthesis (Fig. 6B–D). Additionally, the study found that simvastatin exhibited a dose-dependent biphasic effect on angiogenesis, characterized by low-dose angiogenesis promotion and high-dose anti-angiogenesis within a specific dose range (Fig. 6D) [144]. Compared to BMP-2 and VEGF, simvastatin has the advantages of low cost and long half-life. Moreover, the sustained release of simvastatin from hydrogels can promote the production of endogenous bioactive factors like BMP-2 and VEGF, key regulators of osteogenesis and angiogenesis in bone regeneration, effectively promoting bone tissue regeneration [145].

### 3.2 Infectious pathological microenvironment

Although the porous structure of 3D-printed porous titanium alloy prosthesis can promote host cell adhesion, growth, and proliferation, the porous structure also has a certain likelihood of causing bacterial aggregation, conducive to bacterial growth and proliferation [29]. Some artificial joint prostheses easily adhere to bacteria and form biofilms, promoting bone infections. Therefore, developing new artificial joint prosthesis with dual antibacterial and osteogenesis functions is crucial for arthroplasty in infectious pathological microenvironments. Among numerous antibacterial materials, antibacterial hydrogels are increasingly gaining attention as ideal substitutes for antibiotic treatment of bacterial infectious diseases due to their multiple biological functions and wide adaptability. Additionally, using antibacterial hydrogels as antibacterial coatings on bone prosthesis can enhance their antibacterial performance, providing more options for artificial joint prosthesis in infectious pathological microenvironments. Below, we will detail studies on promoting bone growth and osseointegration in infectious pathological microenvironments using porous titanium alloy prosthesis modified with hydrogels loaded with different components.

#### 3.2.1 Autologous antibacterial hydrogels

CS is a linear polysaccharide composed of D-glucosamine and N-acetyl-D-glucosamine and their derivatives [146], with excellent biodegradability and biocompatibility, exerting antibacterial effects through direct contact with bacteria [147]. CS's antibacterial mechanism mainly relies on the protonation of its structural amino groups in weakly acidic solutions, disrupting bacterial cell membranes through electrostatic interactions, thereby inhibiting bacterial growth [148]. Tian et al. [149] designed a dual-functional, thermosensitive, and injectable hydrogel composed of CS, quaternary ammonium chitosan (QCS), and nHA, exhibiting excellent biocompatibility, antibacterial properties, and osteogenic differentiation-promoting capabilities. This hydrogel showed outstanding *in vivo* therapeutic effects in infected bone defect rabbit models, offering a promising material design and comprehensive one-step treatment strategy for infected bone defects. Additionally, CS can be used as hydrogels for bone tissue engineering or composite coatings on prosthesis to promote sterilization and enhance the ability of the prosthesis to cope with infectious pathological microenvironments.

#### 3.2.2 Nanosilver (NAg) particles

**Fig. 7:**
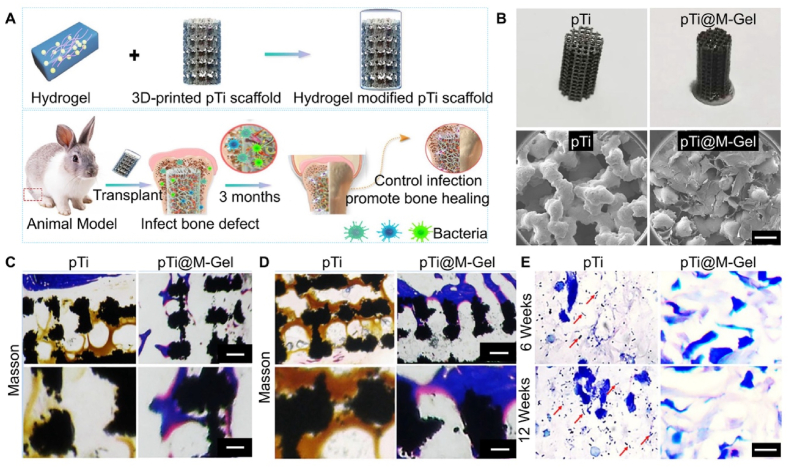
Pathological infection microenvironment. Reproduced with permission from Ref. [29] (Copyright 2020, Journal of Tissue Engineering). (A) Schematic of the combination of supramolecular hydrogel with dual antibacterial and osteoinductive functions and 3D-printed PTI for treating infectious bone defects. (B) Macroscopic appearance of PTI scaffolds and PTI scaffolds modified with supramolecular hydrogel, and the microstructure of PTI scaffolds and PTI scaffolds modified with supramolecular hydrogel (scale bar = 1 mm). (C) Representative Masson staining images at 6 weeks post-prosthesisation. (D) Representative Masson staining images at 12 weeks post-prosthesisation (black areas indicate PTI scaffolds, blue areas indicate bone, and yellow areas indicate pus; upper image, scale bar = 600 μm; lower image, scale bar = 200 μm). (E) Giemsa staining images of tissue surrounding the scaffold surface (red arrows indicate bacteria; scale bar = 10 μm). (For interpretation of the references to colour in this figure legend, the reader is referred to the Web version of this article.)

Metal and metal oxide nanoparticles have broad application prospects in antibacterial treatments, capable of killing bacteria or inhibiting pathogen production through multiple mechanisms. Nanosilver particles, as broad-spectrum antibacterial agents, can counter pathogen invasion through multiple mechanisms and effectively reduce resistance [150]. Compared to silver ions, NAg has better penetration and retention effects, making NAg more effective in antibacterial applications [151]. Therefore, Qiao et al. [22] first constructed a composite system of AgNPs-loaded hydrogel and 3D-printed porous titanium alloy prosthesis to promote bone regeneration in infectious pathological microenvironments (Fig. 7A and B). Nanoparticles themselves have been proven to bind to cells; migrate into cells; and disrupt proteins, genetic material, and membranes, hence causing cell death. The NAg released locally as the hydrogel degrades can interact with negatively charged bacterial cell walls, increasing permeability. NAg can trigger reactive oxygen species (ROS) generation within cells, leading to cell death through oxidative stress, membrane lipid damage, and peroxidation [152,153]. Reactive oxygen species (ROS) are molecules that contain one or more oxygen atoms, including both free radicals and non-radical forms, such as superoxide anions, hydrogen peroxide, and hydroxyl radicals [154]. In bone tissue engineering, ROS play a dual role: in appropriate amounts, they can promote the proliferation, differentiation, and repair of bone cells, while excessive ROS can lead to oxidative damage, impairing bone repair and osseointegration [155,156]. Therefore, the regulation of ROS balance is crucial for optimizing bone repair and tissue regeneration in bone tissue engineering. Additionally, ROS can interact with bacterial nucleic acids and proteins. *In vitro* experiments demonstrated that the composite prosthesis had good biocompatibility, promoting BMSCS proliferation and osteogenic differentiation while inhibiting bacterial growth (Fig. 7E). *In vivo* experiments further confirmed that this prosthesis effectively promoted bone regeneration while possessing effective antibacterial capabilities, promising to be an effective method for addressing infectious bone defects (Fig. 7C and D).

#### 3.2.3 Antibiotics

Infection control is crucial for promoting bone regeneration in infectious pathological microenvironments. For patients at risk of infection, systemic antibiotic therapy is commonly used clinically. However, systemic antibiotic administration cannot achieve effective local antibacterial concentrations, thereby failing to inhibit potential infections. By contrast, local antibiotic administration can release high concentrations of antibiotics, effectively preventing infections and avoiding the drawbacks of systemic antibiotic use. Doxycycline (Dox) is used clinically as a broad-spectrum antibiotic in orthopedic surgery and can inhibit bacterial protein synthesis and ribosomes. Additionally, Dox can stimulate bone formation and has certain inhibitory effects on inflammation and osteoclastogenesis. Therefore, Zhao et al. [20] loaded Dox into a chitosan-gelatin hydrogel and combined it with 3D-printed porous titanium alloy prosthesis to create antibacterial composite prosthesis. The mixed system exhibited sustained antibacterial effects and generated anti-adhesion properties, having strong clearance capabilities against microorganisms. The composite system maintained a barrier against soft tissue growth, while releasing antibiotics, crucial for promoting bone regeneration in infectious pathological microenvironments. Additionally, CS itself is a polycationic polysaccharide with both antibacterial and osteogenesis-enhancing properties [156]. When combined with other antibacterial substances in hydrogel form and coated on 3D-printed porous titanium alloy scaffolds, it can enhance the composite system's infection-fighting ability [61]. The composite system maintained a barrier against soft tissue growth while releasing antibiotics, crucial for promoting bone regeneration in infectious pathological microenvironments.

Methicillin-resistant *Staphylococcus aureus* (MRSA) is the main pathogen in peri-prosthetic infections, with strong resistance to common clinical antibiotics and high pathogenicity and rapid spread; it is referred to as a “superbug.” Vancomycin is a cationic glycopeptide antibiotic acting on the carboxyl termini of the D-Ala-D-Ala precursors of bacterial cell walls, inhibiting cell wall synthesis and exerting antibacterial effects. It is clinically effective against infections caused by drug-resistant bacteria, particularly MRSA and methicillin-resistant *Staphylococcus epidermidis*. Additionally, research indicates that vancomycin has better biocompatibility with OB and bone cells than other commonly used antibiotics like ciprofloxacin and tobramycin [157]. Therefore, Huang et al. [158] chose to combine carboxymethyl chitosan-hyaluronic acid hydrogels loaded with vancomycin and 3D-printed porous titanium alloy to address prosthesis-related infections caused by *S. aureus* and MRSA. The slow degradation of carboxymethyl chitosan-hyaluronic acid hydrogels *in vivo* enabled the sustained release of vancomycin, avoiding cell toxicity caused by explosive release. Vancomycin allowed controlled loading and release, significantly inhibiting MRSA adhesion and colonization on prosthesis, preventing prosthesis-related infections without affecting MC 3T3-E1 cell adhesion, proliferation, and osteogenic differentiation. This local drug delivery method allowed high local antibiotic concentrations to penetrate biofilms and bone tissue without harming other body tissues, avoiding damage to vital organs like the liver and kidneys. The composite prosthesis, combining vancomycin-loaded carboxymethyl chitosan-hyaluronic acid hydrogels and 3D-printed porous titanium alloy, is a new type of titanium alloy prosthesis with both antibacterial and osteogenic properties, significant for preventing prosthesis-related infections and promoting bone regeneration in infectious pathological microenvironments.

### 3.3 Osteoporotic pathological microenvironment

Osteoporosis is a systemic bone disease characterized by reduced bone mass, impaired bone quality, and decreased bone strength, leading to increased bone fragility and susceptibility to fractures [159,160]. Increased bone resorption and insufficient bone formation are the direct causes of osteoporosis, leading to bone-related defects and fractures. Promoting bone regeneration and repair in osteoporotic conditions is a critical clinical issue. On the one hand, excessive bone resorption and insufficient bone formation in osteoporotic conditions lead to slow healing of bone defects. On the other hand, the osteogenic capacity of the body is poor under osteoporotic pathological conditions, interfering with bone-prosthesis integration. Additionally, previous studies have shown that 8 weeks after osteoporotic fractures, local bone resorption remains highly active, while collagen fiber formation and mineralization are relatively inactive, further delaying new bone formation and remodeling. Therefore, surface modification of prosthesis is essential to improve the imbalance of the osteogenic-osteoclastic microenvironment around the prosthesis in osteoporotic patients.

#### 3.3.1 Drugs

##### 3.3.1.1 Bisphosphonates

**Fig. 8:**
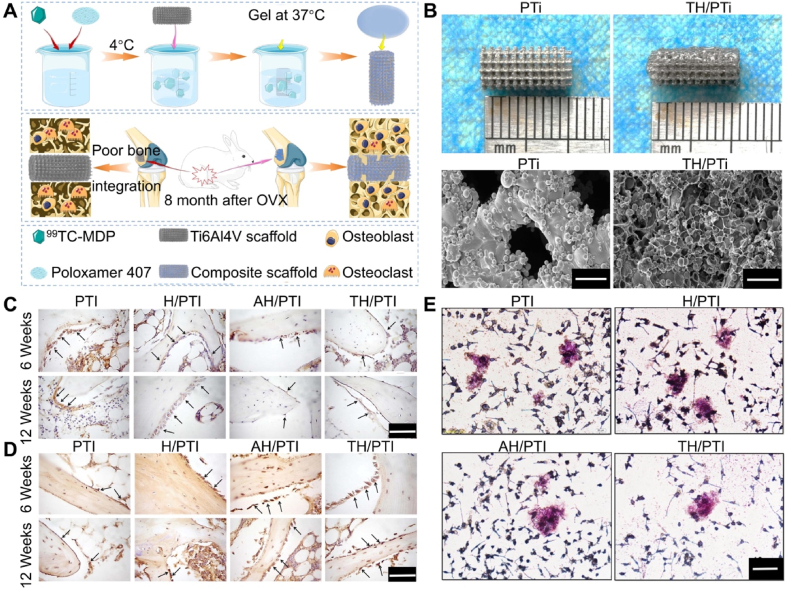
Osteoporotic pathological microenvironment. Reproduced with permission from Ref. [43] (Copyright 2021, Materials & Design). (A) Schematic of the 99 TC-MDP hydrogel composite system (TH/PTI) for treating osteoporotic bone defects. (B) Macroscopic appearance and microstructure of PTI scaffolds and TH/PTI scaffolds (scale bar = 500 μm). (C) Expression of RANKL in the bone tissue surrounding the scaffolds at 6 and 12 weeks post-prosthesisation (scale bar = 100 μm). (D) Expression of OPG in the bone tissue surrounding the scaffolds at 6 and 12 weeks post-prosthesisation (scale bar = s100 μm). (E) Effect of the scaffolds on OC formation in different groups (scale bar = 40 μm).

Bisphosphonates (BPs), as osteoclast (OC) inhibitors, are the first-line drugs for treating osteoporosis. BPs inhibit bone resorption by inhibiting the expression of OC inducer RANKL and promoting the secretion of RANKL competitive inhibitor osteoprotegerin (OPG), preventing RANKL and RANK interaction, thus inhibiting OC function and excessive bone resorption. Unlike mechanisms acting on OC, BPs can affect bone resorption by influencing protein activity in OB. Therefore, BPs promote OB function while indirectly limiting OC formation and bone resorption. However, systemic administration methods such as oral or intravenous administration pose high risks, potentially leading to complications like jaw osteonecrosis. Compared to systemic administration, local application of bisphosphonates (BPs) allows for targeted delivery directly to the bone defect site, enhancing local bioavailability and avoiding systemic metabolism, thus improving therapeutic efficacy [161]. By inhibiting osteoclast activity and reducing bone resorption, local administration effectively promotes bone repair and optimizes bone regeneration. Moreover, local delivery minimizes systemic side effects and offers precise, targeted treatment, making it a safe and effective strategy for the repair of osteoporotic bone defects [162]. Zoledronic acid (ZOL), a third-generation BP drug, primarily exerts anti-osteoporotic effects by acting on OC. Bai et al. [40] loaded ZOL into Poloxamer 407 hydrogels and combined them with 3D-printed porous titanium alloy prosthesis to evaluate the osteogenic capacity of this composite system under osteoporotic conditions. ZOL can achieve sustained drug release with the slow degradation of the hydrogel, maintaining optimal local ZOL concentrations. The *in vitro* drug release curve showed that ZOL could maintain concentrations of 10^−7^–10^−8^ M for a long time. Previous studies indicated that these ZOL concentrations could promote OC apoptosis, upregulate *OB* gene expression, and downregulate *OC* gene expression. Existing studies have shown that for osteoporotic patients requiring intraosseous prosthesis treatment such as arthroplasty, using low-dose BPs to treat osteoporosis does not increase the risk of prosthesis failure. Regardless of the administration method (oral vs. intravenous), there is no significant decrease in peri-prosthesis bone levels, and prosthesis survival rates remain relatively high [163,164]. Therefore, this mixed system can promote BMSCS osteogenesis and inhibit OC formation. Additionally, in OVX rabbit distal femoral defect models, this mixed system accelerated bone regeneration and enhanced osseointegration. Technetium methylene diphosphonate (99Tc-MDP) is a novel anti-osteoporotic drug, a chelate of 99Tc and methylene diphosphonate, inhibiting inflammation and regulating immunity, delaying and repairing bone erosion, and promoting osteogenesis. Studies have shown that 99Tc-MDP has significant anti-osteoporotic effects, with low local application concentrations promoting human OB proliferation and osteogenic differentiation. Compared to traditional BPs, 99Tc-MDP can inhibit the secretion of various osteoclast-related immune factors, better regulating the osteoporotic microenvironment. Cui et al. [43] constructed a composite system of 99Tc-MDP-loaded hydrogel combined with 3D-printed porous titanium alloy prosthesis to regulate the imbalanced osteogenic-osteoclastic microenvironment around the bone-prosthesis interface under osteoporotic conditions (Fig. 8A and B). Utilizing Poloxamer 407 hydrogel, 99Tc-MDP could maintain good drug release, inhibiting OC formation through the RANKL/OPG signaling pathway (Fig. 8E) and promoting OB differentiation, significantly promoting bone regeneration under osteoporotic conditions (Fig. 8C and D). Additionally, this system could control the microstructure of the hydrogel to regulate degradation speed, achieving controlled and sustained drug release. Loading BP drugs into hydrogels and combining them with 3D-printed porous titanium alloy prosthesis can form a bio-camouflaging composite system. Additionally, BP drugs in the composite system can be released sustainably and predictably, promoting bone marrow mesenchymal stem cell proliferation, significantly upregulating osteogenic gene expression, and inhibiting OC formation, thereby promoting bone regeneration and enhancing the mechanical stability of the prosthesis in osteoporosis. Therefore, the composite system composed of 3D-printed porous titanium alloy prosthesis and hydrogels provides a new and effective strategy for joint replacement surgery under osteoporotic pathological conditions.

##### 3.3.1.2 Rapamycin

**Fig. 9:**
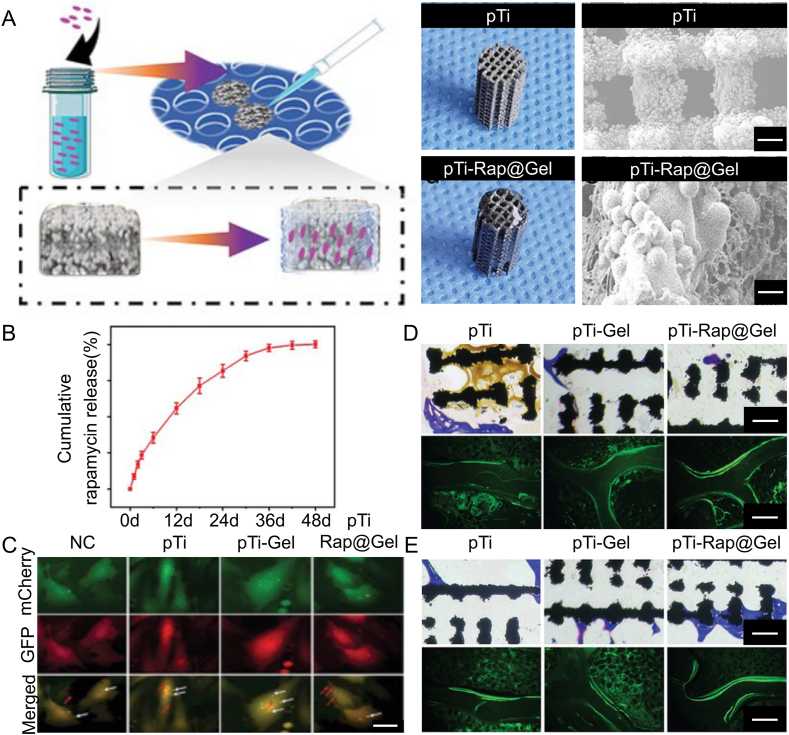
Osteoporotic pathological microenvironment. Reproduced with permission from Ref. [44] (Copyright 2022, Adv. Healthcare Mater). (A) Schematic of the engineering multifunctional hydrogel-integrated 3D-printed bioactive prosthetic interfaces for osteoporotic osseointegration (B) Macroscopic appearance and microstructure of pTI scaffolds and pTI-Rap@Gel scaffolds (upper row of images, scale bar = 400 μm; lower row of images, scale bar = 100 μm). (C) BacLight dead/live staining of MRSA (scale bar = 10 μm). (D) Representative histological images in the middle region of bone defects by Masson staining.

Rapamycin is a macrolide antibiotic that inhibits the mTOR signaling pathway, reducing cell proliferation, protein synthesis, and autophagy, affecting various physiological and pathological processes [165]. In osteoporosis treatment, rapamycin has potential applications as it can promote osteogenic differentiation of BMSCs by regulating autophagy and inhibiting OC formation and activity, reducing bone resorption, and enhancing osseointegration [166]. Based on these properties, Li et al. [44] loaded rapamycin into polysaccharide hydrogels and combined them with 3D-printed porous titanium alloy prosthesis to regulate BMSCS autophagy levels and restore cell activity in the osteoporotic microenvironment (Fig. 9A). The composite system could continuously release rapamycin and BMP-2(Fig. 9B), promoting BMSCS proliferation and differentiation while exhibiting good antibacterial activity against *S. aureus* and MRSA (Fig. 9C). More importantly, the composite system significantly improved bone growth and osseointegration at the prosthesis interface in the osteoporotic microenvironment and effectively inhibited infection (Fig. 9D and E). This study provides an effective strategy for reducing complications after joint replacement surgery in osteoporotic patients.

#### 3.3.2 RNA

Gene therapy was proposed for bone regeneration several years ago. The therapeutic effect is achieved by maintaining or regulating the expression of therapeutic proteins. Based on negative regulation principles, RNA is used as a drug to precisely target key genes, influencing bone regeneration and remodeling. Transferring RNA encoding osteogenic factors into the body through vectors is currently one of the most advanced gene therapy schemes for promoting bone growth. A single gene can broadly regulate bone formation or resorption. Upregulating the expression levels of *Runx2* or *BMP* may induce extensive new bone regeneration. Collaborative RNAi therapy has been applied to improve local bone quality. RNA can also be integrated into biocompatible prosthesis to promote tissue regeneration applications. Prosthesis can load messenger RNA (mRNA) and mimic endogenous microRNA (miRNA) or siRNA to deplete negative regulators of bone formation. (miRNAs are conserved, endogenously synthesized RNA molecules used for RNAi therapy, targeting mRNA to inhibit transcription or RNA cleavage. miRNAs have been reported for osteogenesis, with previous studies proving that upregulating miR-20a expression enhances BMSCs differentiation. Therefore, Liu et al. [167] constructed a 3D-printed porous titanium alloy composite prosthesis with hydrogels loaded with BMSCs overexpressing miR-20a to explore its role in osteogenesis and the potential therapeutic effects on osteoporosis. By using extracellular vesicles to load BM-MSCs overexpressing miR-20a into hydrogels, extracellular vesicle retention and stability were increased, protecting vesicles from premature degradation. Three-dimensional confocal laser scanning results showed a uniform distribution of extracellular vesicles in hydrogels, achieving sustained release with hydrogel degradation. Furthermore, the vesicles released *in vivo* effectively prevented miRNA degradation by serum enzymes. The 3D-printed porous titanium alloy hydrogel composite prosthesis loaded with BMSCs overexpressing miR-20a better promoted MSC migration and differentiation than the control group and exhibited better osteogenesis in the osteoporotic SD rat model induced by ovariectomy. Combining RNA interference technology with bone tissue engineering is a future research direction in bone tissue engineering.

#### 3.3.3 Bioactive factors

**Fig. 10:**
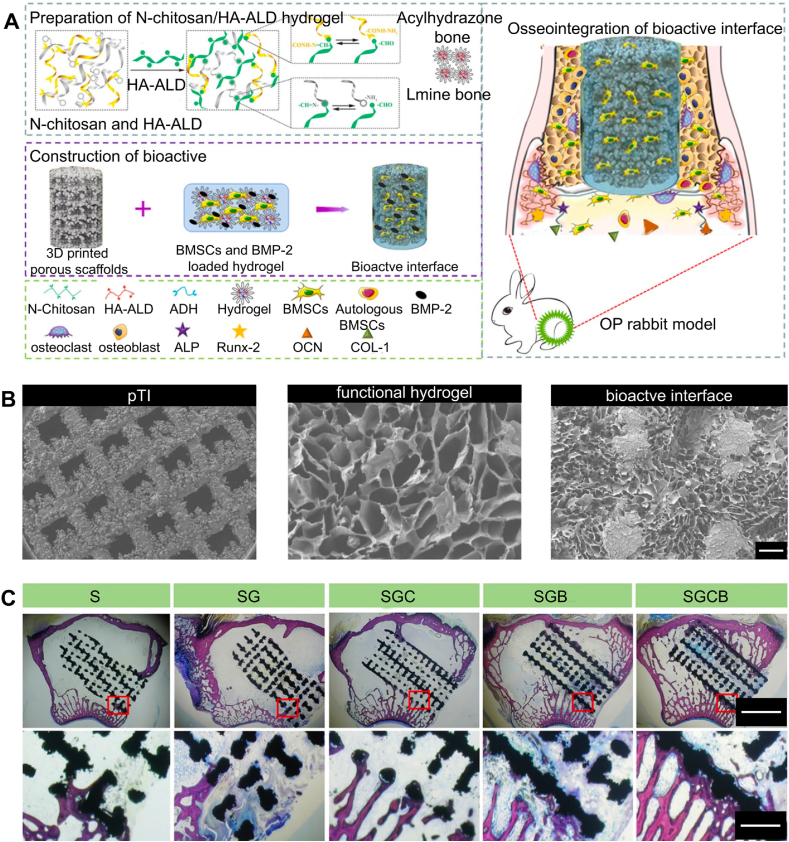
Osteoporotic pathological microenvironment. Reproduced with permission from Ref. [41] (Copyright 2020, Theranostics). (A) Schematic of the enhanced osseointegration of 3D supramolecular bioactive interface through regulation of the osteoporotic microenvironment. (B) Macroscopic appearance and microstructure of pTI scaffolds andfunctional hydrogel and bioactive interface (scale bar = 100 μm). (C) Representative histological photos in the region of bone defect by Van–Gieson staining.

BMPs can induce osteogenic differentiation of stem cells *in vitro* and bone regeneration *in vivo*, playing a significant role in treating critical-sized bone defects [168]. BMP-2 is one of the most osteoinductive BMPs, significantly promoting bone regeneration. BMP-2 can bind to BMP receptors on cell membranes, activating the BMP-specific SMAD pathway cascade, and promoting the expression of osteogenic-related genes to exert osteogenic effects [169]. Existing research has demonstrated good bone regeneration effects with BMP-2 for osteoporotic bone defect regeneration [170]. Under osteoporotic conditions, the imbalance of the osteogenic-osteoclastic microenvironment leads to greater bone resorption than bone formation, severely affecting bone regeneration [171]. Therefore, Bai et al. [41] encapsulated BMP-2 and BMSCs in injectable polysaccharide hydrogels, combining them with 3D-printed porous titanium alloy prosthesis to form bio-camouflaging prosthesis, prosthesised into rabbit osteoporotic bone defect models to evaluate the osseointegration effects of the prosthesis (Fig. 10A and B). Additionally, the polysaccharide hydrogels have good self-healing, injectability, and biodegradability, maintaining BMSCS viability during encapsulation. With biodegradation, BMP-2 achieved sustained release, inducing osteogenic differentiation of bone marrow mesenchymal stem cells, and promoting prosthesis-bone interface integration. Polysaccharide hydrogels loaded with both BMSCS and BMP-2 synergistically induced bone ingrowth, promoting osseointegration of 3D-printed porous titanium alloy prosthesis in osteoporotic bone defects (Fig. 10C).

OPG is a glycoprotein of the tumor necrosis factor (TNF) receptor family, which is considered a factor inhibiting bone resorption. OPG can bind and neutralize RANKL, regulating the RANKL/RANK/OPG system to inhibit osteoclastogenesis and bone resorption. Wang et al. [42] prepared a thermosensitive Poloxamer 407 hydrogel loaded with BMP-2 and OPG, combined with 3D-printed porous titanium alloy prosthesis, to inhibit OC activity and promote osteogenesis. With hydrogel degradation and drug diffusion, BMP-2 and OPG achieved sustained release *in vivo*. In the early prosthesisation stage, rapid drug release because of drug diffusion released high concentrations of BMP-2 and OPG at bone defects, providing quick therapeutic effects while alleviating disease. In the late prosthesisation stage, BMP-2 and OPG are released slowly with hydrogel degradation, maintaining drug concentrations at defect sites. Additionally, the sustained release of BMP-2 and OPG in the composite prosthesis significantly improved bone ingrowth and osseointegration at osteoporotic bone defects. This study indicates that Poloxamer 407 hydrogel-modified 3D-printed porous titanium alloy prosthesis loaded with BMP-2 and OPG can potentially promote osseointegration in joint replacement surgery for osteoporotic patients, providing a new strategy for reducing complications and improving surgical outcomes.

Platelet-rich plasma (PRP) is a platelet suspension extracted and concentrated from the patient's blood, increasing platelet concentration to 3–5 times that of whole blood through centrifugation, rich in PDGF, TGF-β, and VEGF, among other growth factors and cytokines. PRP accelerates BMSCS proliferation, inducing osteogenic differentiation and new bone formation, enhancing blood supply through angiogenesis, and providing necessary nutrients and oxygen for new bone formation and maturation. In orthopedics, PRP is widely used in fracture healing, joint repair, spinal fusion, and prosthesis integration, significantly improving fracture healing speed, cartilage regeneration, bone graft success rates, and prosthesis stability, providing a safe and effective biological therapy for orthopedic diseases. Therefore, Qiao et al. [172] combined PRP with 3D-printed porous titanium alloy scaffolds through lyophilization to enhance osseointegration in osteoporotic patients. The results indicated that the growth factors and cytokines in PRP significantly promoted BMSCS proliferation and differentiation, enhancing angiogenesis, and improving osseointegration in osteoporotic conditions. Compared to scaffolds without PRP coating, PRP-coated scaffolds exhibited better biocompatibility and bone bonding capacity in both *in vivo* and *in vitro* experiments. The study demonstrated PRP's important role in promoting cell proliferation, differentiation, and angiogenesis, providing an effective strategy for joint replacement surgery in osteoporotic patients, and potentially reducing postoperative complications.

#### 3.3.4 Cells

In recent years, stem cell therapy has garnered widespread attention as an alternative strategy to promote prosthesis osseointegration in osteoporotic patients owing to its unique properties [[173], [174], [175]]. However, the primary mechanism of postmenopausal osteoporosis is estrogen deficiency, which leads to ROS accumulation, affecting stem cell osteogenic differentiation and bone formation [176,177]. To improve the osteogenic capacity of osteoporotic patients, Ding et al. [178] developed a ROS-scavenging hydrogel loaded with BMSCs and combined it with 3D-printed porous titanium alloy prosthesis to enhance prosthesis-bone interface osseointegration in osteoporotic patients. The results indicated that the composite system effectively scavenged ROS, protecting BMSCs from oxidative stress damage, and promoting cell proliferation and osteogenic differentiation. Additionally, the ROS-scavenging hydrogel encapsulating BMSCs significantly promoted osseointegration of 3D-printed porous titanium alloy prosthesis in osteoporosis, including scavenging-accumulated ROS, inducing macrophage polarization to the M2 phenotype, inhibiting inflammatory cytokine expression, and improving osteogenic markers (e.g., ALP, RUNX-2, Col-1, BSP, OCN, and OPN). This study provides a new strategy for overcoming the challenge of transplanted stem cells' impaired function in damaged microenvironments and enhancing osseointegration of osteoporotic prosthesis, with broad clinical application prospects.

### 3.4 Tumor pathological microenvironment

**Fig. 11:**
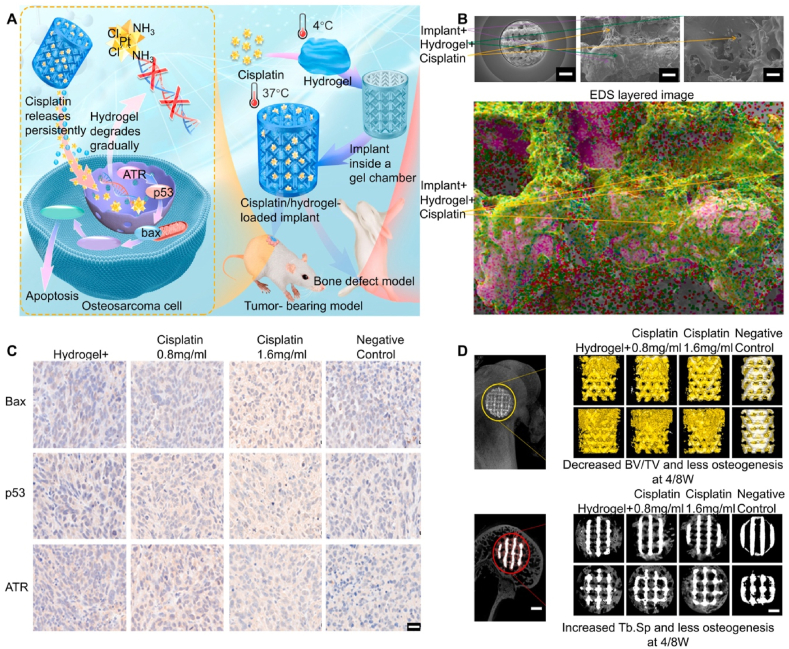
Pathological bone tumor microenvironment. Reproduced with permission from Ref. [37] (Copyright 2021, Bioactive Materials). (A) Schematic of cisplatin/hydrogel 3D-printed Ti6Al4V prosthesis with antitumor and bone repair functions for treating bone tumor-induced bone defects. (B) Characterization of cisplatin/hydrogel 3D-printed Ti6Al4V prosthesis, including scanning electron microscope images and EDS layered images (left image, scale bar = 1 mm; middle image, scale bar = 100 μm; right image, scale bar = 10 μm). (C) Representative immunohistochemical images of tumor sections from mice receiving different treatments showing ATR, p53, and Bax expression (scale bar = 50 μm). (D) Bone integration of the scaffolds in different groups at 4 and 8 weeks post-prosthesisation (scale bar = 1 mm).

Bone tumors pose significant challenges owing to their high malignancy, complexity, invasiveness, and high mortality rates, causing considerable suffering for patients. Clinically, conventional bone tumor treatments primarily include surgical intervention, chemotherapy, and radiotherapy. In recent years, surgical resection combined with perioperative adjuvant chemotherapy has become a common surgical approach for treating bone tumors [179]. Perioperative adjuvant chemotherapy has improved the prognosis of patients with bone tumors. However, traditional intravenous chemotherapy methods can cause numerous adverse reactions, including nephrotoxicity, bone marrow suppression, nausea, vomiting, and low drug concentrations at tumor sites [180,181]. Clinically, local chemotherapy through intraperitoneal, intra-arterial, and intratumoral administration has been used to address these issues [182]. However, given the rapid entry of low molecular-weight chemotherapy drugs like cisplatin into the bloodstream (resulting in short retention time in tumors), the enhancement and prolongation of antitumor effects are not significant [156,183]. Therefore, developing prosthesis with both bone replacement and local chemotherapy drug release functions is crucial for further improving bone tumor patient treatment. Jing et al. [37] utilized the hydrophobic-hydrophilic nature of the PLGA-PEG-PLGA hydrogel to prepare a 3D-printed porous titanium alloy hydrogel composite prosthesis loaded with cisplatin and evaluated its antitumor and bone repair effects (Fig. 11A and B). The *in vitro* drug release experiment results indicated that both groups with different cisplatin doses exhibited sustained release for over 15 days. This demonstrated that PLGA-PEG-PLGA hydrogel-modified 3D-printed porous titanium alloy hydrogel composite prosthesis has certain drug-sustained release capabilities, maintaining effective concentrations of cisplatin at bone defect sites to continuously kill tumor cells. In a human osteosarcoma 143B cell xenograft model established by subcutaneous prosthesis in the right scapula of nude mice, animals treated with prosthesis loaded with hydrogel and cisplatin (prosthesis + hydrogel + cisplatin 0.8 mg/mL and 1.6 mg/mL groups) exhibited dose-dependent antitumor effects, significantly inhibiting tumor growth compared to prosthesis without hydrogel and prosthesis with hydrogel but without cisplatin (Fig. 11C). By directly comparing local drug administration with systemic drug administration, the study highlighted that local drug delivery of cisplatin using cisplatin/hydrogel prosthesis is superior to traditional systemic cisplatin administration in terms of antitumor effects and biosafety. Local chemotherapy delivers higher drug concentrations to the tumor microenvironment while avoiding severe systemic adverse reactions. Additionally, the bone repair efficacy of the composite prosthesis was validated by implanting it into a rabbit bone defect model. The results showed that local cisplatin application reduced osteogenesis in the early stage (4 weeks) but did not affect long-term osteogenesis (8 weeks), with all indicators such as osteogenesis, bone ingrowth, bone bonding, and bone-prosthesis fixation strength fully restored 8 weeks post-surgery, exhibiting acceptable long-term stability (Fig. 11D). The composite system demonstrated good biosafety and significant antitumor potential both *in vitro* and *in vivo*. The composite prosthesis exhibited excellent antitumor and osteogenic capabilities in bone tumor defect repair, with good biocompatibility, providing new ideas and strong clinical translation potential for treating clinical bone tumor defects.

Simvastatin has been widely used for its osteogenic and angiogenic effects. Although the exact potential mechanism of simvastatin's antitumor effects remains unclear [184,185]. Multiple studies have confirmed that statins inhibit the proliferation and metastasis of various tumors to some extent [186,187]. Jing et al. [188] combined simvastatin-loaded PLGA-PEG-PLGA hydrogels with 3D-printed porous titanium alloy prosthesis to achieve both osteogenic and antitumor functions. Compared to pure 3D-printed porous titanium alloy prosthesis, the simvastatin-loaded composite system exhibited enhanced bone formation, growth, and osseointegration, with increased BMP-2 expression, and demonstrated good antitumor activity and biosafety against osteosarcoma. Proteomic sequencing analysis indicated that ferroptosis-related proteins, particularly transferrin (TF) and NADPH oxidase 2 (NOX2), were involved in simvastatin's inhibition of osteosarcoma. It initiates iron-mediated oxidative damage through two main pathways: the intrinsic pathway, involving the activation of iron transporter TF to induce iron overload, and the extrinsic pathway, involving the activation of NOX2 to trigger ROS production for lipid peroxidation [189], thereby achieving antitumor effects.

### 3.5 Immune pathological microenvironment

#### 3.5.1 Rheumatoid pathological microenvironment

Rheumatoid arthritis (RA) is a chronic systemic disease of unknown etiology, often causing joint swelling and pain, cartilage destruction, and ultimately leading to joint deformities and disabilities [190]. For RA patients requiring surgical intervention, the characteristic hypoxic microenvironment may affect bone growth, reducing bone-prosthesis interface integration, and increasing the risk of prosthesis loosening and displacement after joint replacement surgery. Recently, stem cell-based therapies have become a promising alternative for RA management [191,192]. BMSCs, as pluripotent progenitor cells, can differentiate into chondrocytes and OB. For RA joint replacement surgery, BMSCs have been shown to improve bone integration, avoiding postoperative complications such as prosthesis loosening and displacement. Additionally, stem cells can exert anti-inflammatory and immunomodulatory effects by secreting various immune-related bioactive factors, such as cytokines, chemokines, exosomes, and growth factors. Leveraging BMSCs’ osteogenic and immunomodulatory capabilities can improve bone regeneration and inflammatory responses in RA patients, significantly benefiting RA treatment. Zhao et al. [45] encapsulated BMSCs in injectable polysaccharide hydrogels and combined them with 3D-printed porous titanium alloy prosthesis for RA treatment. Polysaccharide hydrogels composed of n-carboxyethyl CS, oxidized hyaluronic acid, and adipic acid dihydrazide (ADH) minimized adverse effects on cultured cells while maintaining long-term integrity. Additionally, owing to dynamic connections, self-healing hydrogels always have injectable properties, enhancing cell-based treatment efficiency, and protecting loaded cells from high shear forces. The prepared polysaccharide hydrogels demonstrated good biocompatibility and biodegradability, achieving complete degradation within 30 days *in vivo*. After loading BMSCs, the composite prosthesis significantly inhibited inflammatory factors, reconstructed damaged cartilage, and promoted subchondral bone formation, exhibiting excellent RA treatment effects.

Compared to BMSCs obtained through bone marrow puncture, adipose-derived stem cells (ADSCs) have significant advantages as they can be obtained minimally invasively from the same patient, avoiding potential immune reactions [[193], [194], [195]]. However, the harsh RA pathological conditions (e.g., excessive protease production, severe inflammation, and high levels of ROS) hinder transplanted ADSCS survival and differentiation [193,196]. Therefore, a suitable carrier is crucial for ADSCs survival and differentiation post-transplantation. Infliximab, a biological therapeutic antibody, inhibits TNF-α, reducing inflammation, disease activity, and cartilage/bone destruction [197]. Additionally, infliximab's amino portion can react with hydroxyl-modified hyaluronic acid in polysaccharide hydrogels, forming imine bonds and further condensation reactions, promoting gel formation [198]. Therefore, Zhao et al. [46] utilized the dynamic reaction between infliximab and modified polysaccharides to prepare an anti-rheumatic drug-based hydrogel combined with 3D-printed porous titanium alloy, used for local drug administration to regulate the pathological microenvironment at transplant sites. The composite system enhanced ADSCS activity under RA conditions, supporting ADSCS proliferation, differentiation, and ECM formation *in vitro*. *In vivo* experiments showed that the bioengineered composite scaffold downregulated inflammatory cytokines, reconstructed damaged cartilage, and promoted subchondral bone repair.

**Fig. 12:**
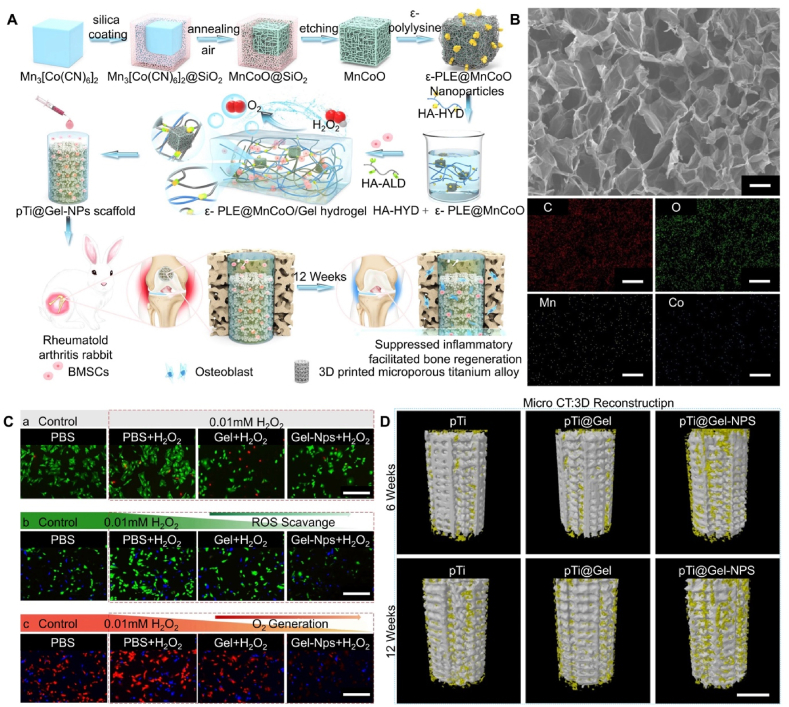
Rheumatoid pathological microenvironment. Reproduced with permission from Ref. [47] (Copyright 2022, Nature Communications). (A) Schematic of prosthesis with ROS scavenging capability for treating rheumatoid bone defects. (B) Scanning electron microscope images of the hydrogel and corresponding elemental maps of the synthesized hydrogel (upper image, scale bar = 200 μm; lower image, scale bar = 500 μm). (C) Effects of different scaffolds on cell viability, ROS scavenging capability, and intracellular oxygen generation (scale bar = 200 μm). (D) Representative 3D reconstructed images of bone regeneration after treatment with different scaffolds at 6 and 12 weeks (scale bar = 5 mm).

Stem cell therapy for RA has garnered significant attention, but treatment efficacy is threatened by ROS accumulation and hypoxia. The infiltration of fibroblast-like synoviocytes and inflammatory factors in the RA synovium increases oxygen consumption, forming a local hypoxic microenvironment [199,200]. Disrupted microvascular structures limit oxygen delivery to the synovium, exacerbating hypoxia [201,202]. Additionally, excessive ROS production under RA conditions remains uncontrolled, further damaging cell metabolism and mitochondrial function [203]. These factors collectively lead to destructive damage to BMSCs in stem cell therapy, severely impacting treatment outcomes. Therefore, to further improve RA treatment efficacy, Zhao et al. [47] combined mesoporous manganese-cobalt oxide (Mn_1.8_Co_1.2_O_4_=MnCoO), a nanozyme with endogenous H_2_O_2_ decomposition and oxygen-generation capabilities, with BMSCs to address ROS accumulation and hypoxia-induced destruction of transplanted BMSCs (Fig. 12A). By using hydrogen peroxide-mimicking nanozymes and dynamically crosslinked natural polymers, the developed nanozyme-enhanced hydrogel encapsulating BMSCs alleviated RA hypoxic and ROS microenvironments, providing a suitable 3D microenvironment for BMSCS proliferation and osteogenesis (Fig. 12C). Combining this with 3D-printed porous titanium alloy prosthesis, the nanozyme-enhanced stem cell hydrogel significantly inhibited inflammatory cytokines and improved the prosthesis interface bone bonding when prosthesised in large-scale bone defects in RA animal models (Fig. 12D).

#### 3.5.2 Diabetic pathological microenvironment

Diabetes is a globally prevalent chronic metabolic disorder characterized by insufficient insulin secretion or insulin resistance, leading to elevated blood glucose levels [204]. As the disease progresses, it can trigger a range of complications that affect multiple organ systems, particularly the skeletal system [205]. Diabetic patients often experience disturbances in bone metabolism, manifested by decreased bone mass, osteoporosis, and an increased risk of fractures [206]. These issues are closely related to the pathological and physiological changes induced by diabetes. One reason for the instability of titanium prosthesis in diabetic patients is increased oxidative stress caused by diabetes in the absence of endogenous antioxidant balance [207]. Previous studies have shown that diabetes-mediated ROS accumulation may adversely affect OB biological performance around titanium alloy surfaces [208,209]. To eliminate ROS generated in diabetic pathological microenvironments affecting titanium prosthesis, Li et al. [210] coated CS hydrogels on 3D-printed porous titanium alloy prosthesis to promote osseointegration under diabetic conditions. CS has been proven to have antioxidant and free radical scavenging activities, eliminating excessive ROS generation in diabetic pathological microenvironments, and reducing functional impairments and apoptosis of OB around the prosthesis [211,212]. Additionally, this composite system promoted AKT phosphorylation by inhibiting ROS, reversing OB dysfunction, and improving cell adhesion, proliferation, and ALP activity. The CS coating significantly improved the impaired biological performance of titanium induced by diabetes through ROS-mediated PI3K/AKT pathway reactivation, providing a novel surface functionalization strategy to improve the clinical performance of titanium prosthesis in diabetic patients.

To mitigate the impaired osteoblast function resulting from abnormal glucose metabolism in the pathological microenvironment of diabetes, Ma et al. [213]developed a Chitosan/Hydroxyapatite (CS/HA) composite coating on the surface of 3D-printed porous titanium alloy prostheses. The objective was to create a bioactive composite system aimed at enhancing the bone integration capacity of 3D-printed porous titanium alloy prostheses under diabetic conditions. The study demonstrated that the CS/HA composite coating (cTi) significantly improved osteoblast functions within diabetic environments, including increased cell adhesion, proliferation, and ALP activity, while concurrently reducing cell apoptosis rates. These enhancements were primarily mediated through the activation of the Wnt/β-catenin signaling pathway. Additionally, *in vivo* experiments using diabetic sheep models further substantiated the advantages of cTi, showing that, compared to uncoated titanium implants, cTi significantly promoted new bone growth and increased bone-implant contact areas. These findings confirm the superior efficacy of cTi in promoting bone integration. In conclusion, the CS/HA composite coating effectively enhances the bone integration capacity of porous titanium alloy prostheses under diabetic conditions by reactivating the Wnt/β-catenin signaling pathway, providing a promising strategy for improving implant success rates in diabetic patients.

## 4 Conclusion and future perspective

Over the past few decades, the rapid development of AM technology has significantly addressed and improved several limitations of traditional titanium alloy prosthesis, making the elastic modulus of the prosthesis similar to that of cancellous bone, thus effectively avoiding stress shielding and bone resorption. However, the biological inertia of titanium alloys limits their further application. Hydrogels can effectively mimic the ECM of natural bone. Coating the surface of titanium alloy prostheses with a hydrogel layer can accurately simulate the physiological environment in which cells survive, providing excellent bio-camouflaging capabilities for the prostheses. This promotes the adhesion, proliferation, and differentiation of osteogenic-related cells, thus effectively addressing the bio-inertness of titanium alloy prostheses. Additionally, hydrogels can serve as carriers for drugs, cells, and bioactive factors, achieving localized release within the body. The composite system of 3D-printed porous titanium alloys and hydrogels not only provides sufficient mechanical support but also offers various components to promote osteogenesis or inhibit osteoclasts depending on different pathological conditions. Combining the advantages of both, this composite system shows significant improvements in angiogenesis, osteogenesis, antibacterial effects, drug delivery, and regulation of the local microenvironment of the prosthesis. In the composite system of 3D-printed porous titanium alloys and hydrogels, the interface bonding between these two materials poses a significant challenge. The adhesion between titanium alloys and hydrogels is often insufficient, leading to potential delamination or detachment during implantation, which can adversely affect the overall mechanical integrity and long-term stability of the system. Particularly after *in vivo* implantation, the interface may degrade further due to mechanical stresses, the accumulation of degradation products, or inflammatory responses, which could impair the prosthesis's bone integration and functionality. Consequently, enhancing the interface adhesion through advanced surface modification techniques or the development of novel interface materials is essential for improving the clinical performance, reliability, and longevity of the composite system in orthopedic applications.

Future developments in this field will continue to drive innovation in orthopedic prosthesis, especially in material optimization, personalized customization, multifunctional composite system development, and interdisciplinary collaboration. The flexibility and precision of 3D printing technology enable personalized customization based on individual patient conditions, perfectly matching the prosthesis with the patient's anatomical structure through the integration of patient imaging data and computer-aided design, thus achieving precision medicine. Additionally, strengthening interdisciplinary collaboration by combining 3D-printing technology with bioengineering, materials science, and drug delivery will develop multifunctional composite systems with properties such as angiogenesis, osteogenesis, ROS clearance, antibacterial, osteoclast inhibition, and antitumor activities. These systems can address issues of insufficient osteogenesis, infection, excessive bone resorption, and tumor recurrence in prosthesis, significantly enhancing therapeutic effects, providing comprehensive treatment plans, and improving bone regeneration and integration of prosthesis in various physiological and pathological microenvironments. Furthermore, long-term follow-up should be conducted after clinical application to evaluate the long-term stability, degradability, and biocompatibility of the prostheses, as well as to identify any potential side effects and complications, thereby optimizing the materials to meet clinical needs. Therefore, future research should focus on these aspects to further enhance the performance of functionalized hydrogel porous titanium alloy systems.

## CRediT authorship contribution statement

**Weimin Zhang:** Writing – original draft, Investigation, Conceptualization. **Jiaxin Zhang:** Writing – review & editing, Data curation. **He Liu:** Validation, Methodology. **Yang Liu:** Supervision, Methodology, Conceptualization. **Xiao Sheng:** Validation, Data curation. **Sixing Zhou:** Validation, Software. **Tiansen Pei:** Software, Methodology. **Chen Li:** Writing – review & editing, Supervision, Funding acquisition, Conceptualization. **Jincheng Wang:** Validation, Methodology, Conceptualization.

## Declaration of competing interest

The authors declare that they have no known competing financial interests or personal relationships that could have appeared to influence the work reported in this paper.

## Data Availability

No data was used for the research described in the article.

## References

[1] Li Z., Wang C., Li C., Wang Z., Yang F., Liu H., Qin Y., Wang J. (2018). What we have achieved in the design of 3D printed metal implants for application in orthopedics? Personal experience and review. Rapid Prototyp. J..

[2] Landgraeber S., Jager M., Jacobs J.J., Hallab N.J. (2014). The pathology of orthopedic implant failure is mediated by innate immune system cytokines. Mediat. Inflamm..

[3] Arcos D., Vallet-Regi M. (2020). Substituted hydroxyapatite coatings of bone implants. J. Mater. Chem. B.

[4] Alvarez K., Nakajima H. (2009). Metallic scaffolds for bone regeneration. Materials.

[5] Xu C., Liu Z., Chen X., Gao Y., Wang W., Zhuang X., Zhang H., Dong X. (2024). Bone tissue engineering scaffold materials: fundamentals, advances, and challenges. Chin. Chem. Lett..

[6] Nune K.C., Kumar A., Murr L.E., Misra R.D. (2016). Interplay between self-assembled structure of bone morphogenetic protein-2 (BMP-2) and osteoblast functions in three-dimensional titanium alloy scaffolds: stimulation of osteogenic activity. J. Biomed. Mater. Res..

[7] Chen Y., Chen X., Hall W.A., Prior P.W., Zhang Y., Paulson E.S., Lang J., Erickson B.A., Li A. (2019). A preferred patient decubitus positioning for MRI-guided online adaptive radiation therapy of pancreatic cancer. Int. J. Radiat. Oncol. Biol. Phys..

[8] Chen Y., Chen X., Hall W.A., Prior P.W., Zhang Y., Paulson E.S., Lang J., Erickson B.A., Li A. (2019). A preferred patient decubitus positioning for MRI-guided online adaptive radiation therapy of pancreatic cancer. Int. J. Radiat. Oncol. Biol. Phys..

[9] Amin Yavari S., van der Stok J., Chai Y.C., Wauthle R., Tahmasebi Birgani Z., Habibovic P., Mulier M., Schrooten J., Weinans H., Zadpoor A.A. (2014). Bone regeneration performance of surface-treated porous titanium. Biomaterials.

[10] Kapat K., Srivas P.K., Rameshbabu A.P., Maity P.P., Jana S., Dutta J., Majumdar P., Chakrabarti D., Dhara S. (2017). Influence of porosity and pore-size distribution in Ti(6)Al(4) V foam on physicomechanical properties, osteogenesis, and quantitative validation of bone ingrowth by micro-computed tomography. ACS Appl. Mater. Interfaces.

[11] Yu X., Wang L., Xia Z., Chen L., Jiang X., Rowe D., Wei M. (2014). Modulation of host osseointegration during bone regeneration by controlling exogenous stem cells differentiation using a material approach. Biomater. Sci..

[12] Liu G., Zhang X., Chen X., He Y., Cheng L., Huo M., Yin J., Hao F., Chen S., Wang P. (2021). Additive manufacturing of structural materials. Mater. Sci. Eng. R Rep..

[13] Wang Z., Wang C., Li C., Qin Y., Zhong L., Chen B., Li Z., Liu H., Chang F., Wang J. (2017). Analysis of factors influencing bone ingrowth into three-dimensional printed porous metal scaffolds: a review. J. Alloys Compd..

[14] Jia Z., Xu X., Zhu D., Zheng Y. (2023). Design, printing, and engineering of regenerative biomaterials for personalized bone healthcare. Prog. Mater. Sci..

[15] Wong K.C. (2016). 3D-printed patient-specific applications in orthopedics. Orthop. Res. Rev..

[16] Popov V.V., Muller-Kamskii G., Kovalevsky A., Dzhenzhera G., Strokin E., Kolomiets A., Ramon J. (2018). Design and 3D-printing of titanium bone implants: brief review of approach and clinical cases. Biomed Eng Lett.

[17] Qian H., Lei T., Hua L., Zhang Y., Wang D.Y., Nan J.Y., Liu W.B., Sun Y., Hu Y.H., Lei P.F. (2023). Fabrication, bacteriostasis and osteointegration properties researches of the additively-manufactured porous tantalum scaffolds loading vancomycin. Bioact. Mater..

[18] Zhao Z.H., Wang M., Shao F., Liu G., Li J.L., Wei X.W., Zhang X.Z., Yang J.H., Cao F., Wang Q.S. (2021). Porous tantalum-composited gelatin nanoparticles hydrogel integrated with mesenchymal stem cell-derived endothelial cells to construct vascularized tissue in vivo. Regenerative Biomaterials.

[19] Davoodi E., Montazerian H., Esmaeilizadeh R., Darabi A.C., Rashidi A., Kadkhodapour J., Jahed H., Hoorfar M., Milani A.S., Weiss P.S. (2021). Additively manufactured gradient porous Ti-6Al-4V hip replacement implants embedded with cell-laden gelatin methacryloyl hydrogels. Acs Applied Materials & Interfaces.

[20] Zhao D.L., Dong H.R., Niu Y.T., Fan W.J., Jiang M.Q., Li K., Wei Q.S., Palin W.M., Zhang Z. (2022). Electrophoretic deposition of novel semi-permeable coatings on 3D-printed Ti-Nb alloy meshes for guided alveolar bone regeneration. Dent. Mater..

[21] Domínguez-Trujillo C., Peón E., Chicardi E., Pérez H., Rodríguez-Ortiz J.A., Pavón J.J., García-Couce J., Galván J.C., García-Moreno F., Torres Y. (2018). Sol-gel deposition of hydroxyapatite coatings on porous titanium for biomedical applications. Surf. Coating. Technol..

[22] Lu Y., Li M., Li L., Wei S., Hu X., Wang X., Shan G., Zhang Y., Xia H., Yin Q. (2018). High-activity chitosan/nano hydroxyapatite/zoledronic acid scaffolds for simultaneous tumor inhibition, bone repair and infection eradication. Mater. Sci. Eng., C.

[23] Komatsu N., Takayanagi H. (2022). Mechanisms of joint destruction in rheumatoid arthritis - immune cell-fibroblast-bone interactions. Nat. Rev. Rheumatol..

[24] Cai Y., Gao T., Fu S., Sun P. (2018). Development of zoledronic acid functionalized hydroxyapatite loaded polymeric nanoparticles for the treatment of osteoporosis. Exp. Ther. Med..

[25] Zhang S., Xing M., Li B. (2018). Biomimetic layer-by-layer self-assembly of nanofilms, nanocoatings, and 3D scaffolds for tissue engineering. Int. J. Mol. Sci..

[26] Zhang S., Cheng X., Yao Y., Wei Y., Han C., Shi Y., Wei Q., Zhang Z. (2015). Porous niobium coatings fabricated with selective laser melting on titanium substrates: preparation, characterization, and cell behavior. Mater. Sci. Eng., C.

[27] Llopis-Grimalt M.A., Arbos A., Gil-Mir M., Mosur A., Kulkarni P., Salito A., Ramis J.M., Monjo M. (2020). Multifunctional properties of quercitrin-coated porous Ti-6Al-4V implants for orthopaedic applications assessed in vitro. J. Clin. Med..

[28] Sheng X., Wang A., Wang Z., Liu H., Wang J., Li C. (2022). Advanced surface modification for 3D-printed titanium alloy implant interface functionalization. Front. Bioeng. Biotechnol..

[29] Qiao S.C., Wu D.L., Li Z.H., Zhu Y., Zhan F., Lai H.C., Gu Y.X. (2020). The combination of multi-functional ingredients-loaded hydrogels and three-dimensional printed porous titanium alloys for infective bone defect treatment. J. Tissue Eng..

[30] Pascalau V., Dindelegan G., Dirzu N., Salantiu A.M., Pavel C., Dudescu M.C., Popa F., Borodi G., Tabaran F., Iuga C.A., Popa C. (2018). Bioactive Ti-base biomaterial with sustained anti-bacterial response for endosseous applications. Reactive Funct. Polym..

[31] Liu J., Qu S., Suo Z., Yang W. (2021). Functional hydrogel coatings. Natl. Sci. Rev..

[32] Cao H., Duan L., Zhang Y., Cao J., Zhang K. (2021). Current hydrogel advances in physicochemical and biological response-driven biomedical application diversity. Signal Transduct Target Ther.

[33] Meng J., Yang X., Huang J., Tuo Z., Hu Y., Liao Z., Tian Y., Deng S., Deng Y., Zhou Z. (2023). Ferroptosis-enhanced immunotherapy with an injectable dextran-chitosan hydrogel for the treatment of malignant ascites in hepatocellular carcinoma. Adv. Sci..

[34] Li S., Pei M., Wan T., Yang H., Gu S., Tao Y., Liu X., Zhou Y., Xu W., Xiao P. (2020). Self-healing hyaluronic acid hydrogels based on dynamic Schiff base linkages as biomaterials. Carbohydr. Polym..

[35] Khajouei S., Ravan H., Ebrahimi A. (2020). DNA hydrogel-empowered biosensing. Adv. Colloid Interface Sci..

[36] Annabi N., Nichol J.W., Zhong X., Ji C., Koshy S., Khademhosseini A., Dehghani F. (2010). Controlling the porosity and microarchitecture of hydrogels for tissue engineering. Tissue Eng. B Rev..

[37] Jing Z.H., Ni R.H., Wang J.D., Lin X.H., Fan D.Y., Wei Q.G., Zhang T., Zheng Y.F., Cai H., Liu Z.J. (2021). Practical strategy to construct anti-osteosarcoma bone substitutes by loading cisplatin into 3D-printed titanium alloy implants using a thermosensitive hydrogel. Bioact. Mater..

[38] Sheng X., Liu H., Xu Y., Wang Z., Zhang W., Li C., Wang J. (2024). Functionalized biomimetic mineralized collagen promotes osseointegration of 3D-printed titanium alloy microporous interface. Mater Today Bio.

[39] Wang Z., Zhao Y., Bai H., Chang F., Yang X., Wang X., Liu J., Wu M., Lin Q., Wang J., Liu H. (2024). Bioactive prosthesis interface compositing variable-stiffness hydrogels regulates stem cells fates to facilitate osseointegration through mechanotransduction. Int. J. Biol. Macromol..

[40] Bai H.T., Cui Y.T., Wang C.Y., Wang Z.H., Luo W.B., Liu Y.Z., Leng Y., Wang J.C., Li Z.H., Liu H. (2020). 3D printed porous biomimetic composition sustained release zoledronate to promote osteointegration of osteoporotic defects. Mater. Des..

[41] Bai H., Zhao Y., Wang C., Wang Z., Wang J., Liu H., Feng Y., Lin Q., Li Z., Liu H. (2020). Enhanced osseointegration of three-dimensional supramolecular bioactive interface through osteoporotic microenvironment regulation. Theranostics.

[42] Wang X., Li Z., Wang Z., Liu H., Cui Y., Liu Y., Ren M., Zhan H., Li Z., Wu M., Wang J. (2021). Incorporation of bone morphogenetic protein-2 and osteoprotegerin in 3D-printed Ti6Al4V scaffolds enhances osseointegration under osteoporotic conditions. Front. Bioeng. Biotechnol..

[43] Cui Y.T., Wang Z.H., Li Z.H., Ji X., Yuan B.M., Sun Y., Peng C.G., Leng Y., Dou M.H., Wang J.C. (2021). Functionalized anti-osteoporosis drug delivery system enhances osseointegration of an inorganic–organic bioactive interface in osteoporotic microenvironment. Mater. Des..

[44] Li Z.H., Zhao Y., Wang Z.H., Ren M., Wang X.G., Liu H., Lin Q., Wang J.C. (2022). Engineering multifunctional hydrogel-integrated 3D printed bioactive prosthetic interfaces for osteoporotic osseointegration. Adv. Healthcare Mater..

[45] Zhao Y., Wang Z., Jiang Y., Liu H., Song S., Wang C., Li Z., Yang Z., Liu H., Wang J. (2019). Biomimetic composite scaffolds to manipulate stem cells for aiding rheumatoid arthritis management. Adv. Funct. Mater..

[46] Zhao Y., Gao C., Liu H., Liu H., Feng Y., Li Z., Liu H., Wang J., Yang B., Lin Q. (2021). Infliximab-based self-healing hydrogel composite scaffold enhances stem cell survival, engraftment, and function in rheumatoid arthritis treatment. Acta Biomater..

[47] Zhao Y., Song S., Wang D., Liu H., Zhang J., Li Z., Wang J., Ren X., Zhao Y. (2022). Nanozyme-reinforced hydrogel as a H(2)O(2)-driven oxygenerator for enhancing prosthetic interface osseointegration in rheumatoid arthritis therapy. Nat. Commun..

[48] Yang X.Y., Chen L.H., Li Y., Rooke J.C., Sanchez C., Su B.L. (2017). Hierarchically porous materials: synthesis strategies and structure design. Chem. Soc. Rev..

[49] Tomatsu I., Peng K., Kros A. (2011). Photoresponsive hydrogels for biomedical applications. Adv. Drug Deliv. Rev..

[50] Li J., Cui X.L., Lindberg G.C.J., Alcala-Orozco C.R., Hooper G.J., Lim K.S., Woodfield T.B.F. (2022). Hybrid fabrication of photo-clickable vascular hydrogels with additive manufactured titanium implants for enhanced osseointegration and vascularized bone formation. Biofabrication.

[51] Wu K., Liu M., Li N., Zhang L., Meng F., Zhao L., Liu M., Zhang Y. (2020). Chitosan-miRNA functionalized microporous titanium oxide surfaces via a layer-by-layer approach with a sustained release profile for enhanced osteogenic activity. J Nanobiotechnology.

[52] Pandya A.D., Overbye A., Sahariah P., Gaware V.S., Hogset H., Masson M., Hogset A., Maelandsmo G.M., Skotland T., Sandvig K., Iversen T.G. (2020). Drug-loaded photosensitizer-chitosan nanoparticles for combinatorial chemo- and photodynamic-therapy of cancer. Biomacromolecules.

[53] Tsai W.B., Chen Y.R., Li W.T., Lai J.Y., Liu H.L. (2012). RGD-conjugated UV-crosslinked chitosan scaffolds inoculated with mesenchymal stem cells for bone tissue engineering. Carbohydr. Polym..

[54] Zhao Z., Gao W., Bai H. (2018). A mineral layer as an effective binder to achieve strong bonding between a hydrogel and a solid titanium substrate. J. Mater. Chem. B.

[55] Yang J.Z., Liu F., Zhou C.S., Li H.J., Yang G.L., Fang S.Y., Lee I.S., Liu Y., Bai H., Chen C. (2023). 3D printed porous titanium filled with mineralized UV-responsive chitosan hydrogel promotes cell proliferation and osteogenesis in vitro. J. Mater. Sci. Technol..

[56] Klouda L. (2015). Thermoresponsive hydrogels in biomedical applications: a seven-year update. Eur. J. Pharm. Biopharm..

[57] Yu S., Zhang X., Tan G., Tian L., Liu D., Liu Y., Yang X., Pan W. (2017). A novel pH-induced thermosensitive hydrogel composed of carboxymethyl chitosan and poloxamer cross-linked by glutaraldehyde for ophthalmic drug delivery. Carbohydr. Polym..

[58] Che Z., Sun Y., Luo W., Zhu L., Li Y., Zhu C., Liu T., Huang L. (2022). Bifunctionalized hydrogels promote angiogenesis and osseointegration at the interface of three-dimensionally printed porous titanium scaffolds. Mater. Des..

[59] Boccaccini A.R., Keim S., Ma R., Li Y., Zhitomirsky I. (2010). Electrophoretic deposition of biomaterials. J R Soc Interface.

[60] Joung Y.S., Ramirez R.B., Bailey E., Adenekan R., Buie C.R. (2017). Conductive hydrogel films produced by freestanding electrophoretic deposition and polymerization at the interface of immiscible liquids. Compos. Sci. Technol..

[61] Ordikhani F., Tamjid E., Simchi A. (2014). Characterization and antibacterial performance of electrodeposited chitosan-vancomycin composite coatings for prevention of implant-associated infections. Mater. Sci. Eng., C.

[62] Waresindo W.X., Luthfianti H.R., Priyanto A., Hapidin D.A., Edikresnha D., Aimon A.H., Suciati T., Khairurrijal K. (2023). Freeze–thaw hydrogel fabrication method: basic principles, synthesis parameters, properties, and biomedical applications. Mater. Res. Express.

[63] Peng Y., Gardner D.J., Han Y. (2011). Drying cellulose nanofibrils: in search of a suitable method. Cellulose.

[64] Koivunotko E., Merivaara A., Niemela A., Valkonen S., Manninen K., Makinen H., Viljanen M., Svedstrom K., Diaz A., Holler M. (2021). Molecular insights on successful reconstitution of freeze-dried nanofibrillated cellulose hydrogel. ACS Appl. Bio Mater..

[65] Liu Z., Xu Z.C., Wang X.Y., Zhang Y.L., Wu Y.Q., Jiang D.Y., Jia R.Z. (2022). Construction and osteogenic effects of 3D-printed porous titanium alloy loaded with VEGF/BMP-2 shell-core microspheres in a sustained-release system. Front. Bioeng. Biotechnol..

[66] Lumelsky N., O'Hayre M., Chander P., Shum L., Somerman M.J. (2018). Autotherapies: enhancing endogenous healing and regeneration. Trends Mol. Med..

[67] Chiu M.C., Li C., Liu X., Yu Y., Huang J., Wan Z., Xiao D., Chu H., Cai J.P., Zhou B. (2022). A bipotential organoid model of respiratory epithelium recapitulates high infectivity of SARS-CoV-2 Omicron variant. Cell Discov.

[68] Zhang S., Liao X., Chen S., Qian W., Li M., Xu Y., Yang M., Li X., Mo S., Tang M. (2022). Large oncosome-loaded VAPA promotes bone-tropic metastasis of hepatocellular carcinoma via formation of osteoclastic pre-metastatic niche. Adv. Sci..

[69] Chen Y.C., Lin R.Z., Qi H., Yang Y., Bae H., Melero-Martin J.M., Khademhosseini A. (2012). Functional human vascular network generated in photocrosslinkable gelatin methacrylate hydrogels. Adv. Funct. Mater..

[70] Ma L.M., Wang X.L., Zhou Y., Ji X.F., Cheng S., Bian D., Fan L., Zhou L., Ning C.Y., Zhang Y. (2021). Biomimetic Ti–6Al–4V alloy/gelatin methacrylate hybrid scaffold with enhanced osteogenic and angiogenic capabilities for large bone defect restoration. Bioact. Mater..

[71] Zhi W., Wang X., Sun D., Chen T., Yuan B., Li X., Chen X., Wang J., Xie Z., Zhu X. (2022). Optimal regenerative repair of large segmental bone defect in a goat model with osteoinductive calcium phosphate bioceramic implants. Bioact. Mater..

[72] Sampath Kumar T.S., Madhumathi K., Rubaiya Y., Doble M. (2015). Dual mode antibacterial activity of ion substituted calcium phosphate nanocarriers for bone infections. Front. Bioeng. Biotechnol..

[73] Hee Soon C., Park S.Y., Kim S., Sang Keun B., Duk Seop S., Ahn M.W. (2008). Effect of different bone substitutes on the concentration of growth factors in platelet-rich plasma. J. Biomater. Appl..

[74] Xu H., Su J., Sun J., Ren T. (2012). Preparation and characterization of new nano-composite scaffolds loaded with vascular stents. Int. J. Mol. Sci..

[75] Feng Y., Wang J., Cao J., Cao F., Chen X. (2024). Manipulating calcium homeostasis with nanoplatforms for enhanced cancer therapy. Exploration.

[76] Hoveidaei A.H., Sadat-Shojai M., Mosalamiaghili S., Salarikia S.R., Roghani-Shahraki H., Ghaderpanah R., Ersi M.H., Conway J.D. (2024). Nano-hydroxyapatite structures for bone regenerative medicine: cell-material interaction. Bone.

[77] Wang Q., Luan J., Zhao Z., Kong W., Zhang C., Ding J. (2023). Dentin-desensitizing biomaterials. Chin. Chem. Lett..

[78] Chu W., Huang Y., Yang C., Liao Y., Zhang X., Yan M., Cui S., Zhao C. (2017). Calcium phosphate nanoparticles functionalized with alendronate-conjugated polyethylene glycol (PEG) for the treatment of bone metastasis. Int J Pharm.

[79] Fan J., Bi L., Wu T., Cao L., Wang D., Nan K., Chen J., Jin D., Jiang S., Pei G. (2012). A combined chitosan/nano-size hydroxyapatite system for the controlled release of icariin. J. Mater. Sci. Mater. Med..

[80] Wang H., Li X., Lai S., Cao Q., Liu Y., Li J., Zhu X., Fu W., Zhang X. (2023). Construction of vascularized tissue engineered bone with nHA-coated BCP bioceramics loaded with peripheral blood-derived MSC and EPC to repair large segmental femoral bone defect. ACS Appl. Mater. Interfaces.

[81] Zhou H., Lee J. (2011). Nanoscale hydroxyapatite particles for bone tissue engineering. Acta Biomater..

[82] Kumar A., Nune K.C., Misra R.D.K. (2018). Design and biological functionality of a novel hybrid Ti!6Al!4V/hydrogel system for reconstruction of bone defects. Journal of Tissue Engineering and Regenerative Medicine.

[83] Kang H.J., Hossain M., Park S.S., Im S.B., Lee B.T. (2021). Microstructures and biological properties of 3D-printed titanium intervertebral spacer with the tri-calcium phosphate loaded demineralized bone matrix hydrogel. Mater. Lett..

[84] Cai L., Lv Y., Yan Q., Guo W. (2024). Cytokines: the links between bone and the immune system. Injury.

[85] Amarasekara D.S., Yu J., Rho J. (2015). Bone loss triggered by the cytokine network in inflammatory autoimmune diseases. J Immunol Res.

[86] Bai Y., Leng Y., Yin G., Pu X., Huang Z., Liao X., Chen X., Yao Y. (2014). Effects of combinations of BMP-2 with FGF-2 and/or VEGF on HUVECs angiogenesis in vitro and CAM angiogenesis in vivo. Cell Tissue Res..

[87] Liu K., Meng C.X., Lv Z.Y., Zhang Y.J., Li J., Li K.Y., Liu F.Z., Zhang B., Cui F.Z. (2020). Enhancement of BMP-2 and VEGF carried by mineralized collagen for mandibular bone regeneration. Regen Biomater.

[88] Lin S., He X., He Y. (2021). Co-culture of ASCs/EPCs and dermal extracellular matrix hydrogel enhances the repair of full-thickness skin wound by promoting angiogenesis. Stem Cell Res. Ther..

[89] Li Y.B., Liu Y.Z., Bai H.T., Li R.H., Shang J., Zhu Z.Q., Zhu L.W., Zhu C.Y., Che Z.J., Wang J.C. (2021). Sustained release of VEGF to promote angiogenesis and osteointegration of three-dimensional printed biomimetic titanium alloy implants. Front. Bioeng. Biotechnol..

[90] Peng Y., Zhuang Y., Liu Y., Le H., Li D., Zhang M., Liu K., Zhang Y., Zuo J., Ding J. (2023). Bioinspired gradient scaffolds for osteochondral tissue engineering. Exploration (Beijing, China).

[91] Zhu L., Liu Y., Wang A., Zhu Z., Li Y., Zhu C., Che Z., Liu T., Liu H., Huang L. (2022). Application of BMP in bone tissue engineering. Front. Bioeng. Biotechnol..

[92] Lavery K., Swain P., Falb D., Alaoui-Ismaili M.H. (2008). BMP-2/4 and BMP-6/7 differentially utilize cell surface receptors to induce osteoblastic differentiation of human bone marrow-derived mesenchymal stem cells. J. Biol. Chem..

[93] <overview_of_bone_morphogenetic_proteins.2.pdf>.

[94] Tian H., Guo A., Li K., Tao B., Lei D., Deng Z. (2022). Effects of a novel self-assembling peptide scaffold on bone regeneration and controlled release of two growth factors. J. Biomed. Mater. Res..

[95] Sun Z., Liu C., Jiang W.G., Ye L. (2020). Deregulated bone morphogenetic proteins and their receptors are associated with disease progression of gastric cancer. Comput. Struct. Biotechnol. J..

[96] Chen L., Jiang W., Huang J., He B.C., Zuo G.W., Zhang W., Luo Q., Shi Q., Zhang B.Q., Wagner E.R. (2010). Insulin-like growth factor 2 (IGF-2) potentiates BMP-9-induced osteogenic differentiation and bone formation. J. Bone Miner. Res..

[97] Pi C.J., Liang K.L., Ke Z.Y., Chen F., Cheng Y., Yin L.J., Deng Z.L., He B.C., Chen L. (2016). Adenovirus-mediated expression of vascular endothelial growth factor-a potentiates bone morphogenetic protein9-induced osteogenic differentiation and bone formation. Biol. Chem..

[98] Hu N., Jiang D., Huang E., Liu X., Li R., Liang X., Kim S.H., Chen X., Gao J.L., Zhang H. (2023). Correction: BMP9-regulated angiogenic signaling plays an important role in the osteogenic differentiation of mesenchymal progenitor cells. J. Cell Sci..

[99] Divband B., Aghazadeh M., Al-Qaim Z.H., Samiei M., Hussein F.H., Shaabani A., Shahi S., Sedghi R. (2021). Bioactive chitosan biguanidine-based injectable hydrogels as a novel BMP-2 and VEGF carrier for osteogenesis of dental pulp stem cells. Carbohydr. Polym..

[100] Shanley L.C., Mahon O.R., Kelly D.J., Dunne A. (2021). Harnessing the innate and adaptive immune system for tissue repair and regeneration: considering more than macrophages. Acta Biomater..

[101] Wang Y.P., Feng Z.J., Liu X., Yang C.F., Gao R., Liu W.S., Ou-Yang W.B., Dong A.J., Zhang C.N., Huang P.S., Wang W.W. (2022). Titanium alloy composited with dual-cytokine releasing polysaccharide hydrogel to enhance osseointegration via osteogenic and macrophage polarization signaling pathways. Regenerative Biomaterials.

[102] Peng Y., Zhuang Y., Zhang Y., Zuo J., Ding J. (2023). Dynamically adaptive scaffolds for cartilage tissue engineering. MedComm – Biomaterials and Applications.

[103] Rostam H.M., Singh S., Salazar F., Magennis P., Hook A., Singh T., Vrana N.E., Alexander M.R., Ghaemmaghami A.M. (2016). The impact of surface chemistry modification on macrophage polarisation. Immunobiology.

[104] Cha B.H., Shin S.R., Leijten J., Li Y.C., Singh S., Liu J.C., Annabi N., Abdi R., Dokmeci M.R., Vrana N.E. (2017). Integrin-mediated interactions control macrophage polarization in 3D hydrogels. Adv Healthc Mater.

[105] Zou M., Sun J., Xiang Z. (2021). Induction of M2-type macrophage differentiation for bone defect repair via an interpenetration network hydrogel with a GO-based controlled release system. Adv Healthc Mater.

[106] Qin S., Niu Y., Zhang Y., Wang W., Zhou J., Bai Y., Ma G. (2024). Metal ion-containing hydrogels: synthesis, properties, and applications in bone tissue engineering. Biomacromolecules.

[107] Xiong A., He Y., Gao L., Li G., Liu S., Weng J., Wang D., Zeng H. (2021). The fabrication of a highly efficient hydrogel based on a functionalized double network loaded with magnesium ion and BMP2 for bone defect synergistic treatment. Mater. Sci. Eng., C.

[108] Janarthanan G., Noh I. (2021). Recent trends in metal ion based hydrogel biomaterials for tissue engineering and other biomedical applications. J. Mater. Sci. Technol..

[109] Saidak Z., Marie P.J. (2012). Strontium signaling: molecular mechanisms and therapeutic implications in osteoporosis. Pharmacol. Ther..

[110] Alsharif S.B., Wali R., Vanyo S.T., Andreana S., Chen K., Sheth B., Swihart M.T., Dziak R., Visser M.B. (2023). Strontium-loaded hydrogel scaffolds to promote gingival fibroblast function. J. Biomed. Mater. Res..

[111] Ren W.-H., Xin S., Yang K., Yu Y.-B., Li S.-M., Zheng J.-J., Huang K., Zeng R.-C., Yang X.-X., Gao L. (2022). Strontium‐doped hydroxyapatite promotes osteogenic differentiation of bone marrow mesenchymal stem cells in osteoporotic rats through the CaSR‐JAK2/STAT3 signaling pathway. Advanced NanoBiomed Research.

[112] Zhao T., Chu Z., Ma J., Ouyang L. (2022). Immunomodulation effect of biomaterials on bone formation. J. Funct. Biomater..

[113] Marin E., Fusi S., Pressacco M., Paussa L., Fedrizzi L. (2010). Characterization of cellular solids in Ti6Al4V for orthopaedic implant applications: trabecular titanium. J. Mech. Behav. Biomed. Mater..

[114] Lovati A.B., Lopa S., Talo G., Previdi S., Recordati C., Mercuri D., Segatti F., Zagra L., Moretti M. (2015). In vivo evaluation of bone deposition in macroporous titanium implants loaded with mesenchymal stem cells and strontium-enriched hydrogel. J. Biomed. Mater. Res. B Appl. Biomater..

[115] Staiger M.P., Pietak A.M., Huadmai J., Dias G. (2006). Magnesium and its alloys as orthopedic biomaterials: a review. Biomaterials.

[116] Romani A.M. (2011). Cellular magnesium homeostasis. Arch. Biochem. Biophys..

[117] Salandova M., van Hengel I.A.J., Apachitei I., Zadpoor A.A., van der Eerden B.C.J. (2021). Fratila-apachitei LE: **inorganic agents for enhanced angiogenesis of orthopedic biomaterials**. Adv Healthc Mater.

[118] Saris N.-E.L., Mervaala E., Karppanen H., Khawaja J.A., Lewenstam A. (2000). Magnesium: an update on physiological, clinical and analytical aspects. Clin. Chim. Acta.

[119] Zhang X., Huang P., Jiang G., Zhang M., Yu F., Dong X., Wang L., Chen Y., Zhang W., Qi Y. (2021). A novel magnesium ion-incorporating dual-crosslinked hydrogel to improve bone scaffold-mediated osteogenesis and angiogenesis. Mater. Sci. Eng., C.

[120] Fu X., Li Y., Huang T., Yu Z., Ma K., Yang M., Liu Q., Pan H., Wang H., Wang J., Guan M. (2018). Runx2/Osterix and zinc uptake synergize to orchestrate osteogenic differentiation and citrate containing bone apatite formation. Adv. Sci..

[121] Huang X., Huang D., Zhu T., Yu X., Xu K., Li H., Qu H., Zhou Z., Cheng K., Wen W., Ye Z. (2021). Sustained zinc release in cooperation with CaP scaffold promoted bone regeneration via directing stem cell fate and triggering a pro-healing immune stimuli. J. Nanobiotechnol..

[122] Qi S., He J., Zheng H., Chen C., Jiang H., Lan S. (2019). Zinc supplementation increased bone mineral density, improves bone histomorphology, and prevents bone loss in diabetic rat. Biol. Trace Elem. Res..

[123] Qu X., Yang H., Yu Z., Jia B., Qiao H., Zheng Y., Dai K. (2020). Serum zinc levels and multiple health outcomes: implications for zinc-based biomaterials. Bioact. Mater..

[124] Song Y., Wu H., Gao Y., Li J., Lin K., Liu B., Lei X., Cheng P., Zhang S., Wang Y. (2020). Zinc silicate/nano-hydroxyapatite/collagen scaffolds promote angiogenesis and bone regeneration via the p38 MAPK pathway in activated monocytes. ACS Appl. Mater. Interfaces.

[125] Lv N., Zhou Z., Hong L., Li H., Liu M., Qian Z. (2024). Zinc-energized dynamic hydrogel accelerates bone regeneration via potentiating the coupling of angiogenesis and osteogenesis. Front. Bioeng. Biotechnol..

[126] Kumar A., Sood A., Singhmar R., Mishra Y.K., Thakur V.K., Han S.S. (2022). Manufacturing functional hydrogels for inducing angiogenic-osteogenic coupled progressions in hard tissue repairs: prospects and challenges. Biomater. Sci..

[127] Xu C., Kang Y., Guan S., Dong X., Jiang D., Qi M. (2023). Iron-based metal–organic framework as a dual cooperative release system for enhanced vascularization and bone regeneration. Chin. Chem. Lett..

[128] Lee E.J., Jain M., Alimperti S. (2021). Bone microvasculature: stimulus for tissue function and regeneration. Tissue Eng Part B Rev.

[129] Li Y.H., Zhang Q.Z., Liu Z.T., Fu C.F., Ding J.X. (2024). Microenvironments-modulated biomaterials enhance spinal cord injury therapy. Adv. Funct. Mater..

[130] Walsh D.P., Raftery R.M., Chen G., Heise A., O'Brien F.J., Cryan S.A. (2019). Rapid healing of a critical-sized bone defect using a collagen-hydroxyapatite scaffold to facilitate low dose, combinatorial growth factor delivery. J Tissue Eng Regen Med.

[131] Wang Z., Wang W., Zhang X., Cao F., Zhang T., Bhakta Pokharel D., Chen D., Li J., Yang J., Xiao C. (2022). Modulation of osteogenesis and angiogenesis activities based on ionic release from Zn-Mg alloys. Materials.

[132] Kfoury Y., Scadden David T. (2015). Mesenchymal cell contributions to the stem cell niche. Cell Stem Cell.

[133] Mavrogenis A., Dimitriou R., Parvizi J., Babis G.C. (2009). Biology of implant osseointegration. J. Musculoskelet. Neuronal Interact..

[134] Insua A., Monje A., Wang H.-L., Miron R.J. (2017). Basis of bone metabolism around dental implants during osseointegration and peri-implant bone loss. J. Biomed. Mater. Res..

[135] Carano R.A.D. (2003). Angiogenesis and bone repair. Drug Discov. Today.

[136] Naoto Koike DF., Oliver Gralla, Au Patrick, Schechner Jeffrey S., Rakesh K. (2004). Jain **Creation of long-lasting blood vessels**. nature.

[137] Toohey K.S., Sottos N.R., Lewis J.A., Moore J.S., White S.R. (2007). Self-healing materials with microvascular networks. Nat. Mater..

[138] Kim K.A., Shin Y.J., Kim J.H., Lee H., Noh S.Y., Jang S.H., Bae O.N. (2012). Dysfunction of endothelial progenitor cells under diabetic conditions and its underlying mechanisms. Arch Pharm. Res. (Seoul).

[139] Kong L., Wang Y., Wang H., Pan Q., Zuo R., Bai S., Zhang X., Lee W.Y., Kang Q., Li G. (2021). Conditioned media from endothelial progenitor cells cultured in simulated microgravity promote angiogenesis and bone fracture healing. Stem Cell Res. Ther..

[140] Zhao H., Shen S., Zhao L., Xu Y.L., Li Y., Zhuo N.Q. (2021). 3D printing of dual-cell delivery titanium alloy scaffolds for improving osseointegration through enhancing angiogenesis and osteogenesis. BMC Muscoskel. Disord..

[141] Huang Y., He B., Wang L., Yuan B., Shu H., Zhang F., Sun L. (2020). Bone marrow mesenchymal stem cell-derived exosomes promote rotator cuff tendon-bone healing by promoting angiogenesis and regulating M1 macrophages in rats. Stem Cell Res. Ther..

[142] Maeda T., Matsunuma A., Kurahashi I., Yanagawa T., Yoshida H., Horiuchi N. (2004). Induction of osteoblast differentiation indices by statins in MC3T3-E1 cells. J. Cell. Biochem..

[143] Tan J., Fu X., Sun C.G., Liu C., Zhang X.H., Cui Y.Y., Guo Q., Ma T., Wang H., Du G.H. (2016). A single CT-guided percutaneous intraosseous injection of thermosensitive simvastatin/poloxamer 407 hydrogel enhances vertebral bone formation in ovariectomized minipigs. Osteoporos. Int..

[144] Liu H., Li W., Liu C., Tan J., Wang H., Hai B., Cai H., Leng H.J., Liu Z.J., Song C.L. (2016). Incorporating simvastatin/poloxamer 407 hydrogel into 3D-printed porous Ti6Al4V scaffolds for the promotion of angiogenesis, osseointegration and bone ingrowth. Biofabrication.

[145] Zhang W., Sun C.G., Zhu J.X., Zhang W.F., Leng H.J., Song C.L. (2020). 3D printed porous titanium cages filled with simvastatin hydrogel promotes bone ingrowth and spinal fusion in rhesus macaques. Biomater. Sci..

[146] Choi C., Nam J.-P., Nah J.-W. (2016). Application of chitosan and chitosan derivatives as biomaterials. J. Ind. Eng. Chem..

[147] Jia W.T., Zhang X., Luo S.H., Liu X., Huang W.H., Rahaman M.N., Day D.E., Zhang C.Q., Xie Z.P., Wang J.Q. (2010). Novel borate glass/chitosan composite as a delivery vehicle for teicoplanin in the treatment of chronic osteomyelitis. Acta Biomater..

[148] Wang J., Zhuang S. (2022). Chitosan-based materials: preparation, modification and application. J. Clean. Prod..

[149] Tian Y., Cui Y., Ren G., Fan Y., Dou M., Li S., Wang G., Wang Y., Peng C., Wu D. (2024). Dual-functional thermosensitive hydrogel for reducing infection and enhancing bone regeneration in infected bone defects. Mater Today Bio.

[150] Knetsch M.L.W., Koole L.H. (2011). New strategies in the development of antimicrobial coatings: the example of increasing usage of silver and silver nanoparticles. Polymers.

[151] Liu H., Wang C., Li C., Qin Y., Wang Z., Yang F., Li Z., Wang J. (2018). A functional chitosan-based hydrogel as a wound dressing and drug delivery system in the treatment of wound healing. RSC Adv..

[152] Chen Y., Wang Z., Xu M., Wang X., Liu R., Liu Q., Zhang Z., Xia T., Zhao J., Jiang G. (2014). Nanosilver incurs an adaptive shunt of energy metabolism mode to glycolysis in tumor and nontumor cells. ACS Nano.

[153] Wang R., Shi M., Xu F., Qiu Y., Zhang P., Shen K., Zhao Q., Yu J., Zhang Y. (2020). Graphdiyne-modified TiO(2) nanofibers with osteoinductive and enhanced photocatalytic antibacterial activities to prevent implant infection. Nat. Commun..

[154] Signorello M.G., Ravera S., Leoncini G. (2020). Lectin-induced oxidative stress in human platelets. Redox Biol..

[155] Kim M.S., Ahn Y.T., Lee C.W., Kim H., An W.G. (2020). Astaxanthin modulates apoptotic molecules to induce death of SKBR3 breast cancer cells. Mar. Drugs.

[156] Dhar S., Liu Z., Thomale J., Dai H., Lippard S.J. (2008). Targeted single-wall carbon nanotube-mediated Pt(IV) prodrug delivery using folate as a homing device. J. Am. Chem. Soc..

[157] Antoci V., Adams C.S., Hickok N.J., Shapiro I.M., Parvizi J. (2007). Antibiotics for local delivery systems cause skeletal cell toxicity in vitro. Clin. Orthop. Relat. Res..

[158] Huang H.K., Wu Z.H., Yang Z.F., Fan X.X., Bai S.Q., Luo J.S., Chen M.M., Xie X.L. (2022). In vitro application of drug-loaded hydrogel combined with 3D-printed porous scaffolds. Biomed. Mater..

[159] Aghebati-Maleki L., Dolati S., Zandi R., Fotouhi A., Ahmadi M., Aghebati A., Nouri M., Kazem Shakouri S., Yousefi M. (2019). Prospect of mesenchymal stem cells in therapy of osteoporosis: a review. J. Cell. Physiol..

[160] Zhang Y., Chen Y., Sun H., Zhang W., Zhang L., Li H., Huang X., Yang J., Ye Z. (2021). SENP3-Mediated PPARγ2 DeSUMOylation in BM-MSCs potentiates glucocorticoid-induced osteoporosis by promoting adipogenesis and weakening osteogenesis. Front. Cell Dev. Biol..

[161] Liu X., Mao X., Ye G., Wang M., Xue K., Zhang Y., Zhang H., Ning X., Zhao M., Song J. (2022). Bioinspired Andrias davidianus-Derived wound dressings for localized drug-elution. Bioact. Mater..

[162] Che L., Wang Y., Sha D., Li G., Wei Z., Liu C., Yuan Y., Song D. (2023). A biomimetic and bioactive scaffold with intelligently pulsatile teriparatide delivery for local and systemic osteoporosis regeneration. Bioact. Mater..

[163] Stavropoulos A., Bertl K., Pietschmann P., Pandis N., Schiodt M., Klinge B. (2018). The effect of antiresorptive drugs on implant therapy: systematic review and meta-analysis. Clin. Oral Implants Res..

[164] Jung R.E., Al-Nawas B., Araujo M., Avila-Ortiz G., Barter S., Brodala N., Chappuis V., Chen B., De Souza A., Almeida R.F. (2018). Group 1 ITI Consensus Report: the influence of implant length and design and medications on clinical and patient-reported outcomes. Clin. Oral Implants Res..

[165] Cui Y., Zhang M., Leng C., Blokzijl T., Jansen B.H., Dijkstra G., Faber K.N. (2020). Pirfenidone inhibits cell proliferation and collagen I production of primary human intestinal fibroblasts. Cells.

[166] Xue S., Zhou X., Sang W., Wang C., Lu H., Xu Y., Zhong Y., Zhu L., He C., Ma J. (2021). Cartilage-targeting peptide-modified dual-drug delivery nanoplatform with NIR laser response for osteoarthritis therapy. Bioact. Mater..

[167] Liu W., Huang J.H., Chen F., Xie D., Wang L.Q., Ye C., Zhu Q., Li X., Li X.L., Yang L.L. (2021). MSC-derived small extracellular vesicles overexpressing miR-20a promoted the osteointegration of porous titanium alloy by enhancing osteogenesis via targeting BAMBI. Stem Cell Res. Ther..

[168] Tanaka M., Sato Y., Zhang M., Haniu H., Okamoto M., Aoki K., Takizawa T., Yoshida K., Sobajima A., Kamanaka T. (2017). In vitro and in vivo evaluation of a three-dimensional porous multi-walled carbon nanotube scaffold for bone regeneration. Nanomaterials.

[169] Bai Y., Liu Y., Jin S., Su K., Zhang H., Ma S. (2019). Expression of microRNA-27a in a rat model of osteonecrosis of the femoral head and its association with TGF-β/Smad7 signalling in osteoblasts. Int. J. Mol. Med..

[170] Cheng Z.A., Alba-Perez A., Gonzalez-Garcia C., Donnelly H., Llopis-Hernandez V., Jayawarna V., Childs P., Shields D.W., Cantini M., Ruiz-Cantu L. (2019). Nanoscale coatings for ultralow dose BMP-2-driven regeneration of critical-sized bone defects. Adv. Sci..

[171] Liu W., Wang T., Zhao X., Dan X., Lu W.W., Pan H. (2016). Akermanite used as an alkaline biodegradable implants for the treatment of osteoporotic bone defect. Bioact. Mater..

[172] Qiao S., Sheng Q., Li Z., Wu D., Zhu Y., Lai H., Gu Y. (2020). 3D-printed Ti6Al4V scaffolds coated with freeze-dried platelet-rich plasma as bioactive interface for enhancing osseointegration in osteoporosis. Mater. Des..

[173] Hejazi F., Ebrahimi V., Asgary M., Piryaei A., Fridoni M.J., Kermani A.A., Zare F., Abdollahifar M.-A. (2021). Improved healing of critical-size femoral defect in osteoporosis rat models using 3D elastin/polycaprolactone/nHA scaffold in combination with mesenchymal stem cells. J. Mater. Sci. Mater. Med..

[174] Laguna E., Pérez-Núñez M.I., del Real Á., Menéndez G., Sáinz-Aja J.A., López-Delgado L., Sañudo C., Martín A., Mazorra R., Ferreño D. (2022). Effects of systemic or local administration of mesenchymal stem cells from patients with osteoporosis or osteoarthritis on femoral fracture healing in a mouse model. Biomolecules.

[175] Niu Q., He J., Wu M., Liu J., Lu X., Zhang L., Jin Z. (2022). Transplantation of bone marrow mesenchymal stem cells and fibrin glue into extraction socket in maxilla promoted bone regeneration in osteoporosis rat. Life Sci..

[176] Qin D., Zhang H., Zhang H., Sun T., Zhao H., Lee W.-H. (2019). Anti-osteoporosis effects of osteoking via reducing reactive oxygen species. J. Ethnopharmacol..

[177] Schröder K. (2019). NADPH oxidases in bone homeostasis and osteoporosis. Free Radic. Biol. Med..

[178] Ding W.B., Zhou Q.R., Lu Y.F., Wei Q., Tang H., Zhang D.H., Liu Z.X., Wang G.C., Wu D.J. (2023). ROS-scavenging hydrogel as protective carrier to regulate stem cells activity and promote osteointegration of 3D printed porous titanium prosthesis in osteoporosis. Front. Bioeng. Biotechnol..

[179] Isakoff M.S., Bielack S.S., Meltzer P., Gorlick R. (2015). Osteosarcoma: current treatment and a collaborative pathway to success. J. Clin. Oncol..

[180] Janeway K.A., Grier H.E. (2010). Sequelae of osteosarcoma medical therapy: a review of rare acute toxicities and late effects. Lancet Oncol..

[181] Konishi M., Tabata Y., Kariya M., Suzuki A., Mandai M., Nanbu K., Takakura K., Fujii S. (2003). In vivo anti-tumor effect through the controlled release of cisplatin from biodegradable gelatin hydrogel. J. Contr. Release.

[182] Nagata Y., Araki N., Kimura H., Fujiwara K., Okajima K., Aoki T., Mitsumori M., Sasai K., Hiraoka M., Higuchi T., Fujii S. (2000). Neoadjuvant chemotherapy by transcatheter arterial infusion method for uterine cervical cancer. J. Vasc. Intervent. Radiol..

[183] Ning S., Yu N., Brown D.M., Kanekal S., Knox S.J. (1999). Radiosensitization by intratumoral administration of cisplatin in a sustained-release drug delivery system. Radiother. Oncol..

[184] Grabarek B.O., Boron D., Morawiec E., Michalski P., Palazzo-Michalska V., Pach L., Dziuk B., Swider M., Zmarzly N. (2021). Crosstalk between statins and cancer prevention and therapy: an update. Pharmaceuticals.

[185] Terzi H., Altun A., Sencan M. (2019). In vitro comparison of the cytotoxic effects of statins on U266 myeloma cell line. Indian J. Med. Res..

[186] Simanshu D.K., Nissley D.V., McCormick F. (2017). RAS proteins and their regulators in human disease. Cell.

[187] Joharatnam-Hogan N., Alexandre L., Yarmolinsky J., Lake B., Capps N., Martin R.M., Ring A., Cafferty F., Langley R.E. (2021). Statins as potential chemoprevention or therapeutic agents in cancer: a model for evaluating repurposed drugs. Curr. Oncol. Rep..

[188] Jing Z., Yuan W., Wang J., Ni R., Qin Y., Mao Z., Wei F., Song C., Zheng Y., Cai H., Liu Z. (2024). Simvastatin/hydrogel-loaded 3D-printed titanium alloy scaffolds suppress osteosarcoma via TF/NOX2-associated ferroptosis while repairing bone defects. Bioact. Mater..

[189] Chen X., Kang R., Kroemer G., Tang D. (2021). Broadening horizons: the role of ferroptosis in cancer. Nat. Rev. Clin. Oncol..

[190] Li C.Y., Sun Y.F., Xu W.G., Chang F., Wang Y.N., Ding J.X. (2024). Mesenchymal stem cells-involved strategies for rheumatoid arthritis therapy. Adv. Sci..

[191] Ansboro S., Roelofs A.J., De Bari C. (2017). Mesenchymal stem cells for the management of rheumatoid arthritis: immune modulation, repair or both?. Curr. Opin. Rheumatol..

[192] Liu Z., Tang M., Zhao J., Chai R., Kang J. (2018). Looking into the future: toward advanced 3D biomaterials for stem-cell-based regenerative medicine. Adv. Mater..

[193] González M.A., Gonzalez-Rey E., Rico L., Büscher D., Delgado M. (2009). Treatment of experimental arthritis by inducing immune tolerance with human adipose-derived mesenchymal stem cells. Arthritis Rheum..

[194] Cai L., Dewi R.E., Goldstone A.B., Cohen J.E., Steele A.N., Woo Y.J., Heilshorn S.C. (2016). Regulating stem cell secretome using injectable hydrogels with in situ network formation. Adv. Healthcare Mater..

[195] Lavrador P., Gaspar V.M., Mano J.F. (2020). Mechanochemical patternable ECM-mimetic hydrogels for programmed cell orientation. Adv. Healthcare Mater..

[196] Yeo J., Lee Y.M., Lee J., Park D., Kim K., Kim J., Park J., Kim W.J. (2019). Nitric oxide-scavenging nanogel for treating rheumatoid arthritis. Nano Lett..

[197] Wojdasiewicz P., Poniatowski Ł.A., Nauman P., Mandat T., Paradowska-Gorycka A., Romanowska-Próchnicka K., Szukiewicz D., Kotela A., Kubaszewski Ł., Kotela I. (2018). Cytokines in the pathogenesis of hemophilic arthropathy. Cytokine Growth Factor Rev..

[198] Wei Z., Yang J.H., Liu Z.Q., Xu F., Zhou J.X., Zrínyi M., Osada Y., Chen Y.M. (2015). Novel biocompatible polysaccharide-based self-healing hydrogel. Adv. Funct. Mater..

[199] Kim J., Kim H.Y., Song S.Y., Go S-h, Sohn H.S., Baik S., Soh M., Kim K., Kim D., Kim H.-C. (2019). Synergistic oxygen generation and reactive oxygen species scavenging by manganese ferrite/ceria Co-decorated nanoparticles for rheumatoid arthritis treatment. ACS Nano.

[200] Willemen N.G.A., Hassan S., Gurian M., Li J., Allijn I.E., Shin S.R., Leijten J. (2021). Oxygen-releasing biomaterials: current challenges and future applications. Trends Biotechnol..

[201] Jeon C.H., Ahn J.K., Chai J.Y., Kim H.J., Bae E.K., Park S.H., Cho E.Y., Cha H.S., Ahn K.S., Koh E.M. (2008). Hypoxia appears at pre-arthritic stage and shows co-localization with early synovial inflammation in collagen induced arthritis. Clin. Exp. Rheumatol..

[202] Peters C.L., Morris C.J., Mapp P.I., Blake D.R., Lewis C.E., Winrow V.R. (2004). The transcription factors hypoxia-inducible factor 1α and Ets-1 colocalize in the hypoxic synovium of inflamed joints in adjuvant-induced arthritis. Arthritis Rheum..

[203] Fearon U., Canavan M., Biniecka M., Veale D.J. (2016). Hypoxia, mitochondrial dysfunction and synovial invasiveness in rheumatoid arthritis. Nat. Rev. Rheumatol..

[204] Andersson O., Adams B.A., Yoo D., Ellis G.C., Gut P., Anderson R.M., German M.S., Stainier D.Y. (2012). Adenosine signaling promotes regeneration of pancreatic β cells in vivo. Cell Metab.

[205] Hulme K.D., Yan L., Marshall R.J., Bloxham C.J., Upton K.R., Hasnain S.Z., Bielefeldt-Ohmann H., Loh Z., Ronacher K., Chew K.Y. (2020). High glucose levels increase influenza-associated damage to the pulmonary epithelial-endothelial barrier. Elife.

[206] Fang C.H., Sun C.K., Lin Y.W., Hung M.C., Lin H.Y., Li C.H., Lin I.P., Chang H.C., Sun J.S., Chang J.Z. (2022). Metformin-incorporated gelatin/nano-hydroxyapatite scaffolds promotes bone regeneration in critical size rat alveolar bone defect model. Int. J. Mol. Sci..

[207] Hamada Y., Fujii H., Fukagawa M. (2009). Role of oxidative stress in diabetic bone disorder. Bone.

[208] Feng Y.F., Wang L., Zhang Y., Li X., Ma Z.S., Zou J.W., Lei W., Zhang Z.Y. (2013). Effect of reactive oxygen species overproduction on osteogenesis of porous titanium implant in the present of diabetes mellitus. Biomaterials.

[209] Lee C.-W., Lin H.-C., Wang B.Y.-H., Wang A.Y.-F., Shin R.L.-Y., Cheung S.Y.L., Lee O.K.-S. (2021). Ginkgolide B monotherapy reverses osteoporosis by regulating oxidative stress-mediated bone homeostasis. Free Radic. Biol. Med..

[210] Li X., Ma X.Y., Feng Y.F., Ma Z.S., Wang J., Ma T.C., Qi W., Lei W., Wang L. (2015). Osseointegration of chitosan coated porous titanium alloy implant by reactive oxygen species-mediated activation of the PI3K/AKT pathway under diabetic conditions. Biomaterials.

[211] Anraku M., Kabashima M., Namura H., Maruyama T., Otagiri M., Gebicki J.M., Furutani N., Tomida H. (2008). Antioxidant protection of human serum albumin by chitosan. Int. J. Biol. Macromol..

[212] Xie W., Xu P., Liu Q. (2001). Antioxidant activity of water-soluble chitosan derivatives. Bioorg. Med. Chem. Lett.

[213] Ma X.Y., Feng Y.F., Ma Z.S., Li X., Wang J., Wang L., Lei W. (2014). The promotion of osteointegration under diabetic conditions using chitosan/hydroxyapatite composite coating on porous titanium surfaces. Biomaterials.

